# LINC02273 drives breast cancer metastasis by epigenetically increasing AGR2 transcription

**DOI:** 10.1186/s12943-019-1115-y

**Published:** 2019-12-19

**Authors:** Bingqiu Xiu, Yayun Chi, Lei Liu, Weiru Chi, Qi Zhang, Jiajian Chen, Rong Guo, Jing Si, Lun Li, Jingyan Xue, Zhi-Ming Shao, Zhao-Hui Wu, Shenglin Huang, Jiong Wu

**Affiliations:** 1 Department of Breast Surgery, Key Laboratory of Breast Cancer in Shanghai, Fudan University Shanghai Cancer Center, Shanghai, 200032 China; 2 Department of Oncology, Fudan University Shanghai Medical College, Shanghai, 200032 China; 3 Department of General Surgery, Nanchang University Second Affiliated Hospital, Nanchang, 330006 China; 4 Department of Pathology and Laboratory Medicine, University of Tennessee Health Science Center, Memphis, TN 38163 USA; 5 Center for Cancer Research, University of Tennessee Health Science Center, Memphis, TN 38163 USA; 6 Fudan University Shanghai Cancer Center, Key Laboratory of Medical Epigenetics and Metabolism, Institutes of Biomedical Sciences, Fudan University, Shanghai, China; 7 Collaborative Innovation Center for Cancer Medicine, Shanghai Medical College, Fudan University, Shanghai, 200032 China

**Keywords:** LINC02273, Breast cancer, Metastasis, AGR2, hnRNPL

## Abstract

**Background:**

The majority of breast cancer patients die of metastasis rather than primary tumors, whereas the molecular mechanisms orchestrating cancer metastasis remains poorly understood. Long noncoding RNAs (lncRNA) have been shown to regulate cancer occurrence and progression. However, the lncRNAs that drive metastasis in cancer patients and their underlying mechanisms are still largely unknown.

**Methods:**

lncRNAs highly expressed in metastatic lymph nodes were identified by microarray. Survival analysis were made by Kaplan-Meier method. Cell proliferation, migration, and invasion assay was performed to confirm the phenotype of LINC02273. Tail vein model and mammary fat pad model were used for in vivo study. RNA pull-down and RIP assay were used to confirm the interaction of hnRNPL and LINC02273. Chromatin isolation by RNA purification followed by sequencing (ChIRP-seq), RNA-seq, ChIP-seq, and luciferase reporter assay reveal hnRNPL-LINC02273 regulates AGR2. Antisense oligonucleotides were used for in vivo treatment.

**Results:**

We identified a novel long noncoding RNA LINC02273, whose expression was significantly elevated in metastatic lesions compared to the primary tumors, by genetic screen of matched tumor samples. Increased LINC02273 promoted breast cancer metastasis in vitro and in vivo. We further showed that LINC02273 was stabilized by hnRNPL, a protein increased in metastatic lesions, in breast cancer cells. Mechanistically, hnRNPL-LINC02273 formed a complex which activated AGR2 transcription and promoted cancer metastasis. The recruitment of hnRNPL-LINC02273 complex to AGR2 promoter region epigenetically upregulated AGR2 by augmenting local H3K4me3 and H3K27ac levels. Combination of AGR2 and LINC02273 was an independent prognostic factor for predicting breast cancer patient survival. Moreover, our data revealed that LINC02273-targeting antisense oligonucleotides (ASO) substantially inhibited breast cancer metastasis in vivo.

**Conclusions:**

Our findings uncover a key role of LINC02273-hnRNPL-AGR2 axis in breast cancer metastasis and provide potential novel therapeutic targets for metastatic breast cancer intervention.

## Background

Although the overall survival and prognosis of breast cancer patients have been improved in recent years [32], metastasis is still the leading cause of mortality in breast cancer patients. Patients with metastases have a 5-year survival rate of only 26%, compared to that of 90% in overall breast cancer patients [36]. Thus, it is important to identify underlying molecular mechanisms of breast cancer metastasis and develop new therapeutic targets. Cancer cells, which displayed high metastatic potential, invade locally and migrate to the secondary organ sites to form metastatic niches. Regional lymph nodes (LNs) are usually the primary sites of early metastasis and axillary lymph node metastasis is one of the most important prognostic factors for breast cancer patients [30]. Thus, novel metastasis-promoting genes in breast cancer may be identified through combing the genetic changes between LN metastasis tissues and respective primary tumors.

In human genome, there is only up to 2% of protein-coding genes that are stably transcribed, whereas the vast majority are non-coding RNAs (ncRNAs) [35]. Emerging evidence has shown that ncRNAs, in particular long non-coding RNAs (lncRNAs) and microRNAs (miRNAs), play crucial roles in various types of cancer through regulating coding gene expression and epigenetic signatures [16, 27]. lncRNAs possess diverse biological functions, such as regulation of protein and RNA stability, transcriptional modulation, nuclear scaffold formation, or guiding protein-DNA interaction [38]. Although previous studies have linked several lncRNAs to cancer metastasis [38], regulatory lncRNAs that initiate and promote the transition from primary cancer to metastatic tumors remain elusive.

Here, we identified that lncRNA LINC02273 was significantly upregulated in metastatic LNs compared with primary tumors and could predict poor recurrence free survival in breast cancer patients. Knockout of LINC02273 could curb breast cancer metastasis in vitro and in vivo. Mechanistically, RNA-binding protein hnRNPL, which was also increased in metastatic tissues, bound with CA-repeats in the 3′ region of LINC02273 through its RRM1&2 motif and increased the stability of LINC02273. We further revealed that the hnRNPL-LINC02273 RNP complex could activate oncogene AGR2 transcription through enhancing H3K4me3 and H3K27ac levels around its promoter region. We also verified the potential clinical use of ASOs targeting LINC02273 to inhibit breast cancer metastasis. Altogether, our results demonstrated that LINC02273 played a pivotal role in enhancing breast cancer metastatic potential and could serve as a novel therapeutic target for mitigating breast cancer metastasis.

## Methods

### Tissue samples

Metastatic lymph node samples and paired primary tumors were collected at Fudan University Shanghai Cancer Center. All lymph node metastatic loci and primary tumors were confirmed by H&E staining. Lymph node metastatic loci and primary tumor sites were dissected and subjected to RNA extraction. A series of fresh frozen tissue and clinical data from breast cancer patients was collected at Fudan University Shanghai Cancer Center. Tumor tissue was obtained from patients undergoing surgery and immediately stored at − 80 °C. All the protocols were reviewed and approved by an independent institutional review board at Fudan University, and all patients gave their written informed consent before inclusion in this study. Post-operative chemotherapy and radiation therapy were suggested according to breast cancer guidelines of Fudan University Shanghai Cancer Center.

bc-GenExMiner 3.0 database was used to explore the predictive role of LINC02273 for metastatic events in breast cancer [14]. Five GEO datasets were pooled (GSE6532, GSE9195, GSE19615, GSE17907, E_MTAB_365). Three hundred fifty-one patients were included, and 81 metastatic events were observed.

### Cell lines, transfection and treatments

The HEK293T, MDA-MB-231 and BT549 cell lines were obtained from American Type Culture Collection (ATCC) (Manassas, VA). LM2 was a subline of MDA-MB-231 with high lung metastasis tendency was described previously [33]. All cell lines were genotyped (Genewiz) and tested routinely for Mycoplasma contamination (Vazyme). All cells are cultured in DMEM containing 10% FBS (Life Technologies), 100 U/ml penicillin and 100 μg/ml streptomycin (Invitrogen) at 37 °C in a humidified incubator with 5% CO2. siRNA and 2-O-methyl RNA/DNA antisense oligonucleotides (ASOs), which were modified by changing the five nucleotides at the 5′ and 3′ ends into 2′-O-methyl were synthesized (RiboBio). siRNA and ASOs transfection were performed using Lipofectamine RNAiMAX (Life Technologies), and plasmid were transfected with Lipofectamine 3000 (Life Technologies). In vivo use GapmeR ASOs with same sequences were chemically modified by Ribobio. The modification of GapmeR were described previously [41]. GapmeR ASOs contains a central block of DNA with a wing region of artificial nucleotides exhibiting high affinity for its target RNA. GapmeR ASOs were dissolved and diluted in PBS to obtain the final concentration of 100 mM. For ASOs free uptake assay, 0.5 × 10^6^ cells were seeded in 6-well plate. After 24 h, GapmeR ASOs were added directly to cell cultures. RNA was extracted 48 h after transfection. For actinomycin D RNA stability assay, 1 × 10^6^ cells were seeded in 6-well plate. After 24 h, 5 μg/ml actinomycin D (Apexbio) were added. Cells were treated at the time point as indicated. Sequences of siRNAs and ASOs were listed in Additional file 1: Table S13.

### RNA isolation, RT-PCR and RT-qPCR

Total RNAs from cultured cells were extracted with TRIzol (Life technologies) according to the manufacturer’s protocol. cDNAs were reverse transcribed with Hiscript III Reverse Transcriptase (Vazyme) with oligo (dT) and random hexamers followed by qRT-PCR analysis and applied for PCR/qPCR analysis. Real time quantitative PCR was performed with ChamQ SYBR qPCR Master Mix (Vazyme) and 7900HT Fast Real-Time PCR System or (Applied Biosystems). The relative expression of different sets of genes was quantified to GAPDH mRNA. Primer sequences for RT-qPCR and RT-PCR used were listed in Additional file 1: Table S12.

### Immunoblotting analysis

Cells (1 × 10^6^) were counted and plated in 6-well plate. After 24 to 48 h, cells were rinsed with PBS. Then, 80 μl 1 × SDS Protein Loading Buffer containing protease and phosphatase inhibitors (Bimake) were added to each well of the plate. Cell lysates were scraped down and sonicated with Bioruptor Plus for 3 × 30s with low power. After denaturation at 100 °C for 10 min, the samples were resolved by SDS-PAGE, transferred to PVDF membranes (Millipore). The membranes were blocked with 5% (w/v) skimmed milk in TBS-T for 1 h, then probing with antibodies at 4 °C overnight. Antibodies used in immunoblot includes hnRNPL (sc-32,317, Santa Cruz Biotechnology); AGR2 (66768–1-Ig, Proteintech); GAPDH (HRP-60004). HRP-conjugated secondary antibodies were used for detection.

### Cell proliferation, wound healing and Transwell assays

For proliferation assay, cells were seeded in 96-well flat-bottomed plates with each well containing 3000 cells in 200 μl of culture medium and cultured in ambient environment described above. Plates were imaged by using IncuCyte ZOOM System (Essen Bioscience) at 12-h interval. The growth rate was measured according to confluence change analyzed by IncuCyte software. For wound healing assay, 30,000 cells were seeded in 96-well IncuCyte® ImageLock Plates (Essen Bioscience). After 24 h, a wound was made in each well with WoundMaker™. Plates were imaged as described above with 6-h interval. Wound width was measured and analyzed. For transwell migration assay, a total of 1 × 10^5^ of MDA-MB-231, 5 × 10^4^ BT549, or 1 × 10^5^ LM2 cells were suspended in 200 μl of DMEM without FBS and seeded on the top chamber of 24-well plate-sized Transwell inserts (Corning Falcon). The lower chambers contained DMEM with 20% FBS. After incubation for 8 h, the inserts were fixed and stained with crystal violet. Cells in the upper chamber were removed with cotton swabs. The average confluence of migrated cells was analyzed by ImageJ according to three random fields captured by 100× microscope. Each experiment was conducted in triplicate. Matrigel invasion assays were performed using Matrigel-coated Transwell inserts with the procedure as described above.

### Rapid amplification of cloned cDNA ends (RACE)

The 3′ and 5′ RACE (Rapid amplification of cloned cDNA ends) was performed using the SMARTer RACE kit (Clontech) following the manufacturer’s instruction. RNA was extracted from MDA-MB-231 cells. Primers used for 3′ and 5′ RACE were listed in Additional file 1: Table S12.

### Ectopic expression and gene knockdown by shRNA

LINC02273 1653 nt full length cDNA were synthesized (Genewiz) according to 5′ and 3′ RACE result and cloned into pCDH-CMV-Puro and pcDNA3.1-myc vector. The ORF of hnRNPL, AGR2, ETS1, and STAT3 were cloned from MDA-MB-231 cDNA and inserted into pCDH-CMV-Puro with C-terminus 3 × FLAG tag. Serial mutations of hnRNPL were subcloned and constructed with C-terminus 3 × FLAG tag. The shRNA oligos were annealed and cloned into pLKO.1-Puro vector. Lentivirus was produced by co-transfecting HEK293T cells with desired plasmids together with psPAX2 and pMD2.G. After 72 h, virus was harvested by passing through a 0.45 mm filter. Collected lentivirus was used directly to infect cells with the addition of 8 mg/ml polybrene (Sigma-Aldrich) or stored in − 80 °C. Infected cells were selected with puromycin (Invivogen) at 1 μg/ml.

### Generation of LINC02273 knockout cell lines by CRISPR-cas9

The gRNA oligos were annealed and cloned into the lentiGuide-Puro (Addgene, 52,963). To delete LINC02273, MDA-MB-231 and BT549 cells were transiently transfected with lentiGuide-Puro plasmid carrying two gRNAs and pLentiCas9-Blast (Addgene, 52,963) and selected with 1 μg/ml puromycin and 2 μg/ml blasticidin for 5 days. Cells were then seeded in 96 well plates in low density (1 cell per well) to form single colonies. Cell clones were genotyped by PCR and sequencing to detect the deletions. PCR primers were listed in Additional file 1: Table S12.

### RNA fish

LINC02273 RNA FISH was performed using Stellaris FISH probes as listed in Additional file 1: Table S11 (Biosearch Technologies) according to the manufacturer’s protocol. In brief, MDA-MB-231 cells were grown on coverslips in a 24-well culture plate. Cells were fixed with 4% (w/v) paraformaldehyde in 1 × PBS for 10 min. Fixed cells were permeabilized with 70% ethanol for 1 h at 4 °C. The coverslips were washed one time with Buffer A (Biosearch Technologies) for 5 min at room temperature and incubated with Stellaris RNA FISH Hybridization Buffer (Biosearch Technologies) containing 10% formamide and 250 nM of the LINC02273-specific Stellaris probe labeled with CAL Fluor Red 590 in the dark at 37 °C overnight. The coverslips were then washed once with Wash Buffer A and once with Wash Buffer A containing 0.1 mg/ml DAPI, both washes in the dark at 37 °C for 30 min and mounted onto microscope slides using VECTASHIELD Antifade Mounting Medium (Vector Laboratories). Images were acquired with Leica confocal microscope.

### Subcellular fraction extraction

1 × 10^7^ cells were collected and used for nuclear and cytoplasmic protein extraction using the NE-PER Nuclear and Cytoplasmic Extraction Reagents (Life Technologies). For nuclear and cytoplasmic RNA separation, 1 × 10^6^ cells were collected and extracted using PARIS™ kit (Life Technologies).

### RIP assay

1 × 10^7^ cells were harvested, resuspended in 1 mL lysis buffer followed by 3 × 10 s sonication with high power with an interval of 30 s. After centrifuging at 12,000 rpm for 15 min at 4 °C, the supernatant was pre-cleared with 30 μl Dynabeads Protein G (Invitrogen). The precleared lysates were incubated with 4 μg anti-hnRNPL antibody (Santa Cruz Biotechnology) for 3 h at 4 °C. Then 40 μl Dynabeads Protein G beads (blocked with 1% BSA and 20 mg/ml yeast tRNA for 1 h at 4 °C) were added to the mixture and incubated for another 1 h at 4 °C followed by washing with wash buffer. The RNA-protein complex was eluted with 200 μl TRIzol at room temperature for 10 min. RNA was purified with Direct-zol RNA Microprep (Zymo Research). For qRT-PCR, reverse transcription was performed with Hiscript III Reverse Transcriptase (Vazyme) with oligo (dT) and random hexamers followed by qRT-PCR analysis. Primers were listed in Additional file 1: Table S12.

### Chromatin isolation by RNA purification

Each probe was designed using LGC Biosearch’s web-based designer, biotin-TEG-labeled at its 3′ terminus and divided into odd or even groups. Probes targeted LacZ were used as negative control. For each sample, 4 × 107 MDA-MB-231 cells were used and crosslinked in 1% glutaraldehyde at room temperature for 10 min, and quenched with 125 mM glycine for 5 min. The cells were lysed by Bioruptor plus in ChIRP lysis buffer and sheared to 100–500 bp fragments at 4 °C. Cleared lysates were hybridized with probes in 37 °C in a hybridization oven for 4 h. After washes and elution, RNA and DNA were purified accordingly. qPCR and RT-qPCR were performed as described above. DNA for library construction were performed as described in the library preparation section. The probes used in the ChIRP assay are listed in Additional file 1: Table S11.

### RNA pull-down assay

PCR products were verified by gel electrophoresis and purified by phenol: chloroform extraction. Biotin-labelled RNAs was in vitro transcribed with HiScribe™ T7 Quick High Yield RNA Synthesis Kit (NEB) with Biotin-16-UTP (Roche), treated with RNase-free DNase I on column during RNA purification with RNA Clean & Concentrator-25 (Zymo Research). For each sample, 5 μg RNA was mixed with 1 × 107 cell extract and incubated at 4 °C for 1 h, followed by incubating with Dynabeads M-280 Streptavidin (Invitrogen) at 4 °C overnight. After washes, the pull-down complexes were eluted by denaturation in 1× protein loading buffer for 10 min at 100 °C. The samples were detected by Western blot or proceed to mass spectrometry.

### Mass spectrometry after RNA pull-down

After RNA pull-down, equal amounts of samples pulled down by sense and anti-sense LINC02273 were loaded on SDS-PAGE gel. Then the gel was stained with Fast Silver Stain Kit (Beyotime) according to the manufacturer’s instructions. Specific bands were cut and analyzed by LC-MS/MS (Shanghai Applied Protein Technology, Shanghai, China). Protein identification was retrieved in the human RefSeq protein database (National Center for Biotechnology Information), using Mascot version 2.4.01 (Matrix Science, London, UK).

### Mammosphere-forming assay

Cells were trypsinized, and 3 × 10^4^ cells per well in 2.5 ml of completed MammoCult™ Medium (Stemcell) in 6-well ultralow attachment plates (Corning). After 5–10 days, mammospheres larger than 100 μm were imaged and counted under microscope.

### Chromatin Immunoprecipitation (ChIP)

MDA-MB-231 cell lines (2 × 10^6^ cells per assay) were cross-linked with 1% formaldehyde at RT for 10 min and quenched in 125 mM glycine for 5 min. The cross-linked chromatin was sonicated to generate DNA fragments averaging 200–500 bp in length by Bioruptor plus. Chromatin fragments were immunoprecipitated with antibodies against mouse or rabbit normal IgG (4 μg, Santa cruz), hnRNPL (4 μg, Santa Cruz), H3K27ac (1:200 CST), H3K4me3 (1:200 CST). The precipitated DNA were purified using the ChIP DNA Clean & Concentrator Kits (Zymo Research).

### Luciferase assay

LINC02273 promoter (chr14:24160663–24,161,805, hg38), AGR2 promoter region, and serial truncations were cloned into pGL3-basic vector. Full length LINC02273 and LINC02273 without CA-repeat were cloned into pMIRGLO vector at the 3′ UTR of luciferase ORF. 1 × 10^5^ cells were plated in each well of 24-well plates. On the next day, 250 ng reporter vector, 250 ng ectopic expression vector and 10 ng pRL-SV40 vector were transfected with Lipofectamine 3000 (Invitrogen). For LINC02273 stability assay, 500 ng reporter vector and 20 pmol siRNA were co-transfected with Lipofectamine 3000 (Invitrogen). After 48 h, cells were lysed in 100 μl of passive lysis buffer. The firefly luciferase activity and the Renilla activity were determined by Dual-Luciferase® Reporter Assay System. For each experiment, the firefly luciferase activity was normalized to Renilla activity. The results were expressed as fold induction compared to negative control indicated.

### Microarray analysis

Five pairs of primary tumor and LN metastatic loci were dissected and verified by H&E staining, tumor percentage > 70%. Followed by RNA extraction with TRIzol reagent, samples were analyzed by Shanghai Qiming Biotechnology Co. Ltd. with Affymetrix HTA 2.0.

### Library preparation and deep sequencing

The total RNA samples (1 μg) of MDA-MB-231 WT and LINC02273 KO cells were treated with VAHTS mRNA Capture Beads (Vazyme) to enrich polyA+ RNA before constructing the RNA-seq libraries. RNA-seq libraries were prepared using VAHTS mRNA-seq v2 Library Prep Kit for Illumina (Vazyme) following the manufacturer’s instructions. Briefly, polyA+ RNA samples were fragmented and then used for first- and second-strand cDNA synthesis with random hexamer primers. The cDNA fragments were treated with DNA End Repair Kit to repair the ends, then modified with Klenow to add an A at the 3′ end of the DNA fragments, and finally ligated to adapters. Purified dsDNA was subjected to 12 cycles of PCR amplification, and the libraries were sequenced by Illumina sequencing platform on a 150 bp paired-end run. Sequencing reads from RNA-seq data were aligned using the spliced read aligner HISAT2, which was supplied with the Ensembl human genome assembly (Genome Reference Consortium GRCh38) as the reference genome. Gene expression levels were calculated by the FPKM (fragments per kilobase of transcript per million mapped reads). Gene Set Enrichment Analysis (GSEA) pre-ranked was run on the ranked list using the Molecular Signatures Database (MSigDB) as the gene sets.

Total RNA was isolated with TRIzol reagent. Next, strand-specific RNA-seq libraries were prepared using the NEBNext Ultra Directional RNA Library Prep kit (New England Biolabs, Beverly, MA, USA), according to the manufacturer’s instructions. For the data processing, the raw sequencing reads were aligned to human reference genome (hg38) using the splice-aware aligner HISAT246. Read counts for each gene were normalized into FPKM (Fragments per Kilobase of transcript per Million mapped reads) values. The cutoff of differential gene expression was FDR < 0.05, normalized by the wild type cells. The FPKM fold change of the overlap genes of the two subgroups independent KO cells were used for further analysis.

ChIP and ChIRP DNA libraries were prepared using the NEBNext DNA Library Prep Kit for Illumina (New England Biolabs) according to the manufacturer’s instructions. The raw reads were aligned by Bowtie2 (v2.2.9) with a human reference genome (hg38). The uniquely mapped reads were subjected to the peak calling algorithm, MACS (v1.4.2) with default parameters.

### In vivo assays

Four-week-old female NOD/SCID mice or female athymic BALB/c nude mice were purchased from Shanghai Slack Laboratory animal Co., LTD and housed under SPF conditions at the animal care facility of the Experimental Animal Center of Shanghai University of Traditional Chinese Medicine.

For xenograft models, 1 × 10^6^ LM2 cells were orthotopically injected directly into the inguinal mammary fat pads of mice in 50 μl of sterile PBS (*n* = 8 in each group).

For ASO in vivo treatment experiment, 1 × 10^6^ LM2 cells were orthotopically injected directly into the mammary fat pads of mice in 100 μl of sterile PBS. Two weeks after injection, mice were randomized into three groups (n = 8 in each group). ASOs were delivered according to groups, through tail vein injection, 10 nmol (100 μl, 100 mM ASO in PBS without transfection reagent) each mouse, and twice a week. Tumor were measured by caliper twice a week. The xenografts were fixed in polyformaldehyde for paraffin embedding or frozen for RNA. For tail vein injection model, 1 × 10^5^ LM2 cells were diluted in 100 μl of sterile PBS and injected through tail vein.

To investigate tumor metastasis, 8 weeks after injection, each mouse was intraperitoneally injected 200 mg/kg D-Luciferin. Bioluminescence were analyzed by IVIS system. All experiments were performed in accordance with relevant institutional and national guidelines and regulations of Shanghai Medical Experimental Animal Care Commission.

### Statistics and reproducibility

Statistical analyses were performed using SPSS v.23.0 (SPSS) or Prism GraphPad 8.0. For most of the experiments, independent sample t-tests were used to calculate the *P* values. For bioluminescence, Mann-Whitney tests were used to calculate the *P* values. Spearman correlation analysis was performed to assess the relationship between different factors. Survival curves were plotted using the Kaplan–Meier method and compared using log-rank tests. All statistical analyses were performed using two-tailed *P* values. The experiments, with the representative data shown in the figures, were repeated three times independently with similar results obtained. Statistical details and methods used are indicated in the figure legends, text or methods.

## Results

### Increased LINC02273 correlates with poor clinical outcome in breast cancer

**Fig. 1:**
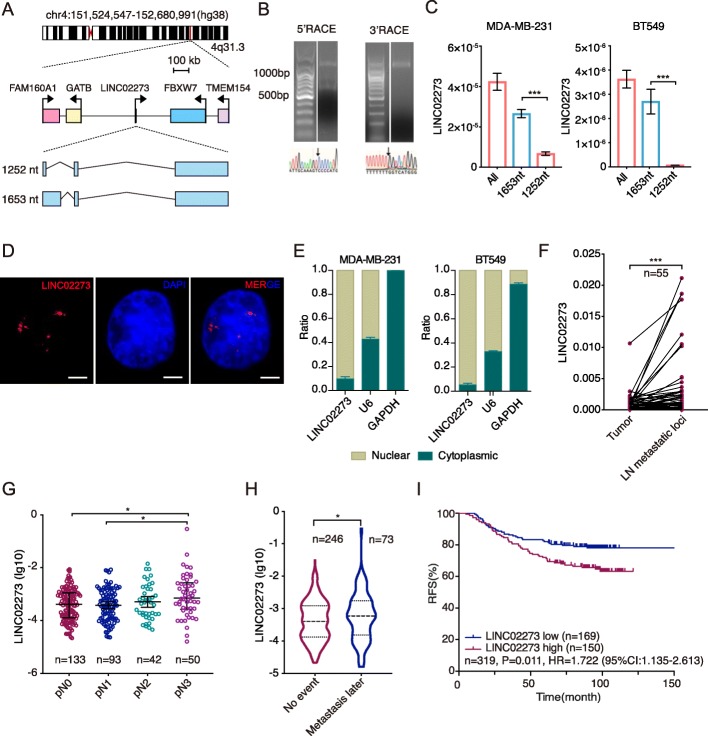
LINC02273 is a prognostic factor in breast cancer. **a** Schematic view of the chromosomal location of LINC02273 and the two isoforms confirmed by RACE-PCR. **b** Representative images from RACE PCR and Sanger sequencing. **c** Relative RNA expression of 1653 nt and 1252 nt isoform in MDA-MB-231 and BT549 cells analyzed by RT-qPCR. Normalized to GAPDH, *n* = 3, biological replicates. **d** LINC02273 localization in MDA-MB-231 revealed by FISH. Blue, DAPI; red, LINC02273 RNA. Scale bar, 5 μm. **e** Cytoplasmic and nuclear fractions were extracted from MDA-MB-231 and BT549 cells and LINC02273 expression was analyzed by RT-qPCR. n = 3, biological replicates. **f** Relative expression of LINC02273 in primary tumor and LN metastatic loci in a 55-paired-sample cohort (Wilcoxon matched-pairs signed rank test). Normalized to GAPDH. **g** RT-qPCR for LINC02273 expression in human breast cancer with different pathological nodal stages (pN, Kruskal-Wallis test). Normalized to GAPDH. **h** Relative expression of LINC02273 in primary tumors from patients suffered metastasis afterward or without metastasis (no event). Mann-Whitney test was used for the statistical analysis. Normalized to GAPDH. **i** Kaplan-Meier recurrence-free survival (RFS) analysis of LINC02273 expression in breast cancer patients (log rank test). Values are expressed as the median with interquartile range in (**g**)-(**h**), mean ± s.e.m. in (**c**). *P < 0.05 and ***P < 0.001

Gene microarray were used to define the transcriptome of metastatic LNs and their matched primary tumors from breast cancer patients (Additional file 1: Figure S1A-B and Table S1). We identified a novel lncRNA LINC02273 was overexpressed in LN metastatic lesions, and inhibition of LINC02273 could impair breast cancer cell migration ability (Additional file 1: Figure S1F- G). LINC02273 is located within human chromosome 4q31.3 (Fig. 1a). Analyses with Coding-Potential Assessment Tool (CPAT) and Coding Potential Calculator (CPC) predicted LINC02273 has minimal coding potential (Additional file 1: Table S2 and S3) [18, 47]. Following 5′ and 3′ rapid amplification of the cDNA ends (RACE) and PCR, we identified two major transcripts of the LINC02273 with 1653-nt and 1252-nt in length (Fig. 1a, b), which share the same transcriptional start site (Fig. 1a). The first exon of 1252-nt isoform is shorter than the 1653-nt due to intron retention. The expression level of 1653-nt isoform is significantly higher than 1252-nt transcript (Fig. 1c; Additional file 1: Figure S1D), and 1653-nt isoform could not spliced into 1252-nt isoform after overexpression (Additional file 1: Figure S1E). As the 1653-nt transcript is the major isoform and shares the same transcriptional start site with 1252-nt transcript, we focused on the 1653-nt isoform of LINC02273 in the following mechanistic studies. RNA in situ hybridization (RNA-ISH) revealed that LINC02273 was mainly localized in nuclei of MDA-MB-231 (Fig. 1d), which was further supported by subcellular fractionation analyses (Fig. 1e).

To further validate the increase of LINC02273 in LN metastases, we examined its expression with quantitative PCR (qRT-PCR) in a breast cancer cohort with lymph node metastasis (*n* = 55). Compared to the primary tumors, LINC02273 was significantly increased in the metastatic loci within LNs (*P* < 0.001, Fig. 1f). Further examining the expression of LINC02273 in primary tumors from a larger breast cancer cohort (*n* = 319) revealed that LINC02273 expression was significantly higher in tumors with positivie lymph nodes (*P = 0.048*). In addition, patients with pN3 lymph nodes metastasis have higher LINC02273 in primary tumors compared with pN1 (*P* = 0.026) or pN0 patients (*P* = 0.026) (Fig. 1g). Furthermore, LINC02273 were highly expressed in tumors of patients who had metastatic cancer later (Fig. 1h). All these data indicate that LINC02273 may drive metastasis in breast cancer patients.

To assess the clinical significance of LINC02273 in breast cancer, we analyzed the relationship between LINC02273 expression level and the clinicopathological characteristics in the 319-cases cohort (Additional file 1: Table S4). Kaplan–Meier survival analysis showed that high LINC02273 expression was correlated with poor recurrence free survival (RFS) (Fig. 1i). In subgroup analysis, ER/PR-positive patients with low LINC02273 expression showed significant RFS benefits (Additional file 1: Figure S1I). The similar survival benefit of low LINC02273 expression was also observed in TNBC and HER2 positive group, although it did not reach statistical significance likely due to smaller sample sizes (Additional file 1: Figure S1J-K). We also evaluate the prognostic value of LINC02273 expression with regression analyses in this cohort. Lymph node metastasis burden and high LINC02273 expression (HR = 1.677, *P* = 0.016) are prognostic predictor for poor RFS (Additional file 1: Table S5). Multivariate analysis further showed that LINC02273 could serve as an independent prognostic factor in breast cancer (HR = 1.543, *P* = 0.045, Additional file 1: Table S6). bc-GenExMiner 3.0 database was used to explore the predictive role of LINC02273 for metastatic events in breast cancer [14]. We found high LINC02273 expression (HR = 1.57, *P* = 0.017) correlated with poor metastasis/recurrence free (MR-free) survival (Additional file 1: Figure S2A). Taken together, these data demonstrate that high LINC02273 expression correlates with poor RFS in breast cancer.

### LINC02273 promotes breast cancer metastasis

**Fig. 2:**
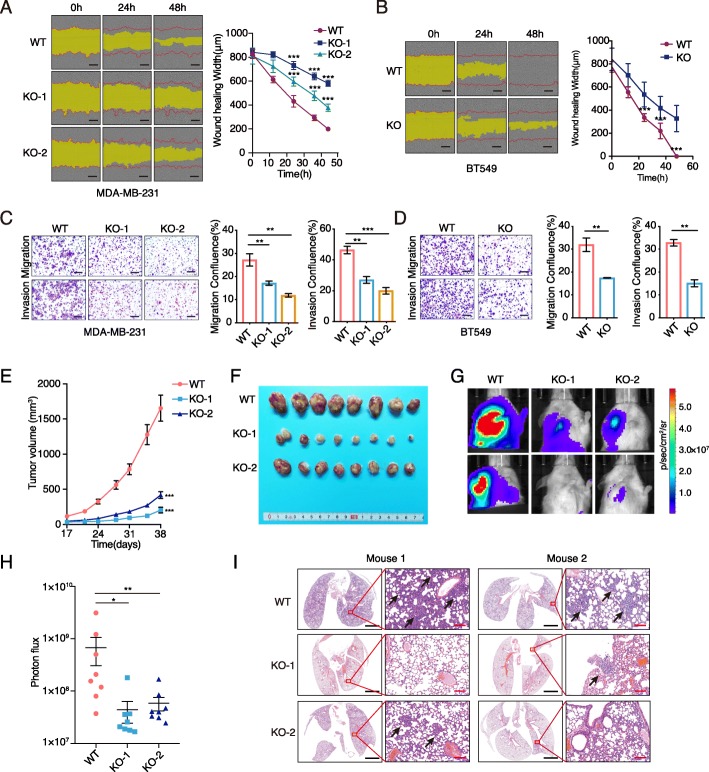
LINC02273 promotes breast cancer metastasis. **a** and **b** Wound healing assay in MDA-MB-231 and BT549 cells. LINC02273 KO cells and WT cells were used. Representative photos and quantitative analysis were shown. Wound width was determined by IncuCyte. Scale bar, 300 μm. *n* = 3, biological replicates. **c** and **d** Transwell migration assay and invasion assay were performed in MDA-MB-231/LINC02273 KO and BT549 cells/LINC02273 KO and the corresponding control cells. Confluence of migrated cells were analyzed by ImageJ. Scale bar, 100 mm. *n* = 3, biological replicates. **e** Tumor volumes were measured in the WT and LINC02273 KO groups in the xenograft mouse model (unpaired Student’s t test was performed on the day 38, *n* = 8 of each group). **f** Representative images of the xenograft tumors isolated from three indicated groups. **g** Representative images of bioluminescence imaging (BLI) on lungs of mice 7 weeks after orthotopic injection of indicated cells. **h** Statistical analysis of lung area photon flux (*n* = 8, means ± s.e.m., Mann-Whitney test). **i** Representative images of H&E staining of lung metastasis. Two representative images were shown from each group. Scale bars: black, 2 mm; pink, 100 μm. **a**-**e**, means ± s.e.m. are shown, and *P* values were determined by independent sample t-tests. *P < 0.05, **P < 0.01, ***P < 0.001. WT, wild type. KO, knockout

To examine the effects of LINC02273 on breast cancer cell proliferation and metastasis, we first analyzed the expression profile of LINC02273 in breast cancer cells. LINC02273 level was relatively higher in cell lines with increased metastatic potential, such as BT549, MDA-MB-231 and MDA-MB-231 lung metastasis cell line LM2 (Additional file 1: Figure S2B). Then, we developed two LINC02273-targting CRISPR-cas9 constructs with distinct guide RNAs (Additional file 1: Figure S2C). Single colonies were selected from breast cancer cells (MDA-MB-231, BT549 and LM2 cells) transduced with LINC02273-targting construct and LINC02273 knock-out efficiency was verified at genomic and transcriptional level (Additional file 1: Figure S2C-E and Fig. S3A, Table S7), and genes closed to LINC02273 were not affected. Loss of LINC02273 significantly decreased the migration and invasion of MDA-MB-231, BT549 and LM2 cells (Fig. 2a-d, Additional file 1: Figure S3E), as well as reduced their proliferation (Additional file 1: Figure S2B). On the contrary, LINC02273 overexpression further enhanced cell migration and invasion of these breast cancer cells (Additional file 1: Figure S2F and Figure S3B-D). The tumor initiation and stemness were not affected after LINC02273 knockout (Additional file 1: Figure S2F).

To further characterize the impact of LINC02273 on cancer metastasis in vivo, orthotopic xenograft models with LM2 wild type (WT) or two clones of LM2 LINC02273-KO cells (KO-1 and KO-2) were used. We found that the growth rate of xenograft tumors was inhibited substantially, and the tumor volumes were significantly reduced when LINC02273 was depleted (Fig. 2e-f). In addition to reduced tumor growth, significantly reduced lung metastasis was observed in mice transplanted with LM2 KO-1 or KO-2 cells, compared to mice with LM2-WT cells (Fig. 2g-i). Taken together, these findings strongly suggested that LINC02273 promoted breast cancer metastasis in vitro and in vivo.

### LINC02273 is stabilized by hnRNPL

**Fig. 3:**
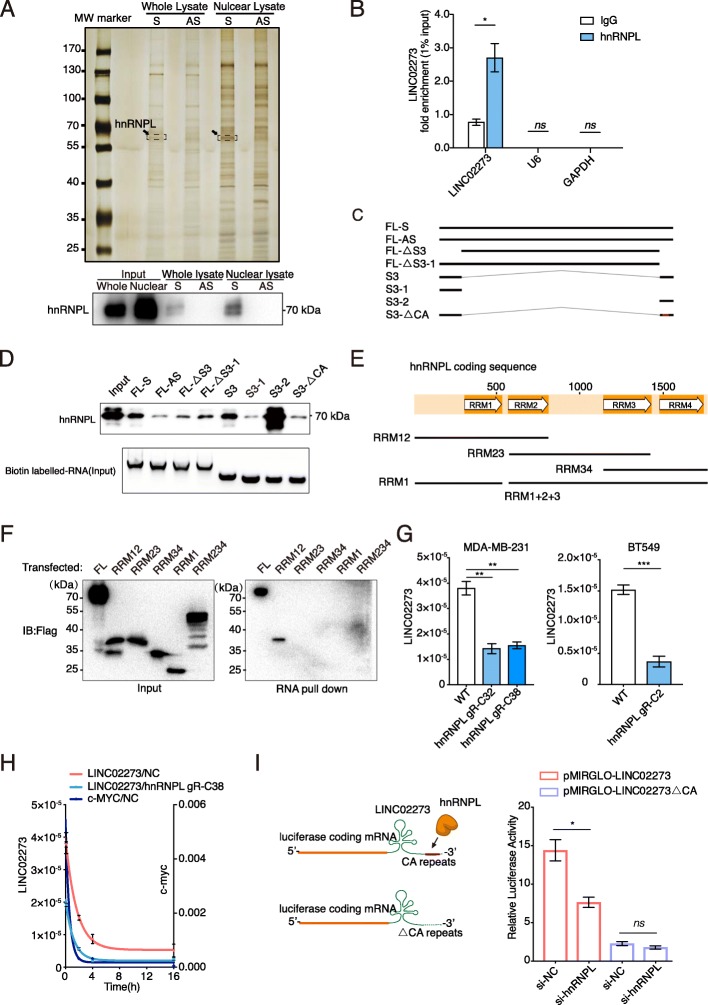
LINC02273 interacts with hnRNPL. **a** Identification of proteins associated with LINC02273. Up: silver staining of RNA pull-down proteins. Whole cell lysates and nuclear fraction were incubated with in vitro transcribed RNA of sense (S) and anti-sense (AS) LINC02273. LINC02273-S enriched bands were analyzed by MS (bands in dash lines), *n* = 3 independent experiment. LIN02273-AS was used as negative control. Bottom: Validation of LINC02273 and hnRNPL interaction by immunoblotting. **b** RIP and RT-qPCR were shown. IgG was used as negative control. *n* = 3, biological replicates. **c** Schematic view of truncated LINC02273. FL (full length)-AS was used as negative control. RNAs corresponding to indicated fragments were biotinylated and incubated with whole cell lysates and subjected to streptavidin pull-down. **d** hnRNPL binding domain on LINC02273. Up, immunoblotting was used to confirm hnRNPL binding. Bottom: Input RNA (3 μg) was shown by native agarose electrophoresis. **e** Schematic view of RNA binding motif of hnRNPL. Up, schematic view of hnRNPL ORF and four RRM domains. Bottom, hnRNPL was truncated according to RRMs. **f** RNA pull-down was performed with ectopically overexpressed truncated hnRNPL with 3 × FLAG in 293 T cells and detected by immunoblotting. *n* = 3, biological replicates. **g** RNA expression of LINC02273 after hnRNPL KO. **h** Half-life of LINC02273 in WT and hnRNPL KO MDA-MB-231 cells treated with 5 μg/mL Actinomycin D. c-myc served as positive control. RNA was extracted and assayed by RT-qPCR. *n* = 3, biological replicates. **i** LINC02273 stability assay. LINC02273 with or without CA-repeat was ligated to the 3′ of luciferase ORF on pmirGLO vector. hnRNPL was knocked down by pooled siRNA. RNA was assayed by RT-qPCR. *n* = 3, biological replicates. For **g**-**h**, RT-qPCR data are normalized to GAPDH. For (**b**) and **g**-**i**, means ± s.e.m. are shown, and *P* values were determined by independent sample t-tests. *P < 0.05, **P < 0.01, ***P < 0.001

To explore how LINC02273 promotes breast cancer metastasis, RNA pull-down assays were performed to identify the protein partners binding to LINC02273. Given that LINC002273 mainly located in cell nucleus, both whole cell lysate and nuclear fraction of MDA-MB-231 cells were incubated with biotinylated LINC02273 transcribed in vitro or its antisense RNA. In both conditions, hnRNPL was identified by mass spectrometry as a major protein in LINC02273 pull-down samples, but not in those from LINC02273-AS (Fig. 3a; Additional file 1: Figure S4A, S4B; Additional file 1: Table S8). The selective binding of hnRNPL to LINC02273 was further confirmed by immunoblotting (Fig. 3a). Reciprocally, hnRNPL RIP analyses of co-purified RNAs from immunoprecipitated hnRNPL confirmed that LINC02273 was significantly enriched in hnRNPL immunoprecipitates compared to other control RNAs (Fig. 3b; Additional file 1: Figure S4C). These results indicate that LINC02273 specifically interacts with hnRNPL in breast cancer cells.

To further map the specific hnRNPL-binding region, a series of deletion mutants of LINC02273 were constructed according to its secondary structure predicted with RNAfold (Additional file 1: Figure S4D) [26]. RNA pull-down of seven deletion mutants of LINC02273 followed by western blot revealed that the region essential for hnRNPL binding was S3–2 (Fig. 3c-d). Previous study reported that hnRNPL bound to CA-repeats sequence of RNA [19]. A 20 base-pair CA stretch was located within the S3–2 region. To validate if this specific CA region is required for hnRNPL binding, CA repeats in S3–2 region were deleted as in mutant S3-△CA. Loss of the CA repeats effectively abrogated LINC02273-hnRNPL binding, confirming the critical role of S3–2 CA-repeats for hnRNPL binding to LINC02273 (Fig. 3d).

Previous studies have reported that hnRNPL have four RNA recognition motifs (RRMs, Fig. 3e), while the RNA binding capacity of hnRNPL is mainly mediated by RRM1, RRM3 and RRM4 motifs [49]. To further examine which motif of hnRNPL interacts with LINC02273, we made hnRNPL truncation mutants and determined their capacity to bind LINC02273 with RNA pull-down assay (Fig. 3e). Although previous study suggested that, functioning as a unit, RRM3/4 is more critical for hnRNPL-RNA binding than RRM1/2 [49], we found that RRM1 and RRM2 together were responsible for hnRNPL binding to LINC02273 (Fig. 3f). These data supported that the CA-repeat within S3–2 region of LINC02273 and RRM1/2 motifs of hnRNPL is required for their interaction.

Interestingly, we noticed that knockdown of hnRNPL reduced LINC02273 mRNA level in MDA-MB-231 and BT549 cells (Fig. 3g; Additional file 1: Figure S4E). Although hnRNPL was reported to have DNA-binding capacity and could affect mRNA transcription [21], luciferase reporter assay showed that overexpression of hnRNPL had little effect on LINC02273 promoter activity, which suggested that hnRNPL may not upregulate LINC02273 through transactivation (Additional file 1: Figure S4F-G). RNA stability could also be affected by RNA binding proteins. We found LINC02273 was relatively stable in MDA-MB-231 cells (Fig. 3h). Interestingly, when RNA transcription was blocked by actinomycin D, the half-life of LINC02273 RNA was significantly decreased after hnRNPL depletion compared to that in control cells. Decrease of hnRNPL expression significantly reduced LINC02273 stability to a level comparable to short-lived MYC mRNA (t_1/2_ 0.5-1 h), indicating hnRNPL stabilizes LINC02273 (Fig. 3h). Accordingly, silencing hnRNPL with siRNA significantly decreased the activity of luciferase reporter gene fused to LINC02273 compared to WT reporter gene (Fig. 3i; Additional file 1: Figure S4H). Moreover, the luciferase activity of reporter gene fused to LINC02273-△CA mutant, which is deficient of hnRNPL binding, was dramatically reduced regardless hnRNPL knock-down (Fig. 3i). All these data suggested that LINC02273 stability was enhanced by hnRNPL binding to the CA-repeats within its S3–2 region.

### LINC02273 stabilizing by hnRNPL promotes breast cancer metastasis

**Fig. 4:**
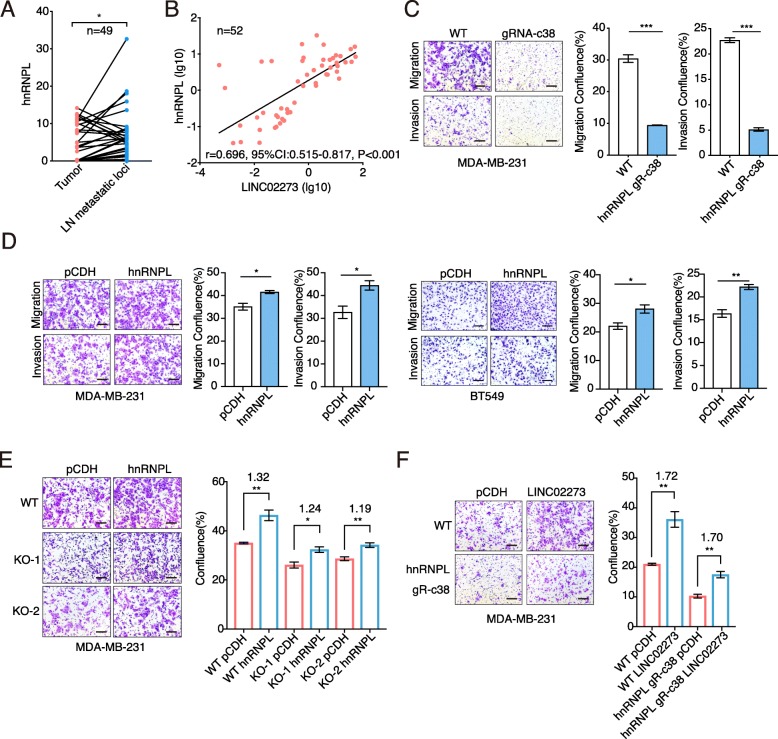
hnRNPL-LINC02273 promotes breast cancer progression. **a** Relative expression of hnRNPL in primary tumor and LN metastatic loci in a 49-patient cohort (Wilcoxon matched-pairs signed rank test). Normalized to GAPDH. **b** Correlation analysis between hnRNPL and LINC02273 in metastatic loci of LN. *n* = 52 samples (Spearman r). **c** Transwell migration and invasion assay after hnRNPL knockdown. **d** Transwell migration and invasion assay after hnRNPL ectopic overexpression in MDA-MB-231 and BT549 cells. Representative images and statistical analysis were shown. Scale bars: 100 μm. *n* = 3, biological replicates. **e** Transwell assay in WT and LINC02273 KO cells with hnRNPL overexpression. Left, representative images. Right, statistical analysis. Average fold change was labeled on the graph. Scale bars: 100 μm. n = 3, biological replicates (**f**) Transwell assay in WT and hnRNPL knockdown cells with LINC02273 overexpression. Left, representative images. Right, statistical analysis. Average fold change was labeled on the graph. Scale bars: 100 μm. n = 3, biological replicates. For (**c**-**f**), means ± s.e.m. are shown, and *P* values were determined by independent sample t-tests. *P < 0.05, **P < 0.01, ***P < 0.001

Previous studies have reported that hnRNPL is involved in mRNA splicing, embryonic development and cancer metastasis [8, 20, 24]. We found, compared to the matched primary tumors, hnRNPL level was increased in metastatic LNs and correlated well with LINC02273 (Fig. 4a-b). Consistently, knockdown of hnRNPL significantly inhibited, while ectopic overexpression of hnRNPL enhanced, breast cancer cell migration and invasion (Fig. 4c-d; Additional file 1: Figure S4I), supporting a metastasis-promoting role of hnRNPL in breast cancer. Considering the effect of hnRNPL on LINC02273 stability, we hypothesized that hnRNPL might exert its pathological effects through stabilizing LINC02273. To this notion, we ectopically expressed hnRNPL in LINC02273 knocked-out cells. The migration of cancer cells was remarkably impaired by LINC02273 knock-out, compared to the WT cells. In accordance, ectopic hnRNPL-enhanced cell migration was significantly reduced in LINC02273-KO cells compared to that in WT cells (Fig. 4e). On the other hand, LINC02273 overexpression in hnRNPL-silenced cells partially restored the cells migration activity which had been reduced by hnRNPL knock-down in MDA-MB-231 (Fig. 4f), demonstrating the significant role of LINC02273 in mediating hnRNPL-promoted metastasis. Taken together, our results strongly support that hnRNPL may promote breast cancer metastasis through stabilizing LINC02273.

### LINC02273 regulates AGR2 through transcriptional activation

**Fig. 5:**
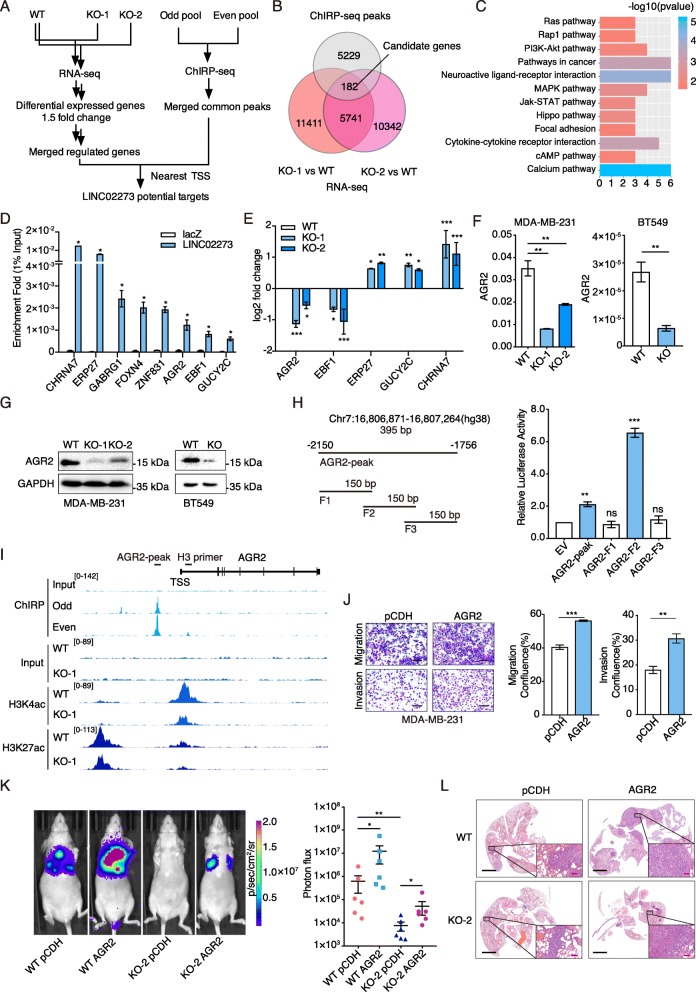
LINC02273 transcriptionally activates AGR2 expression. **a** Flow chart of LINC02273 targets selection. Differential genes were selected by comparing WT to LINC02273 KO MDA-MB-231 cells. LINC02273 DNA binding sites were selected by merging odd and even pool of ChIRP-seq. RNA-DNA binding in − 2000-200 bp to transcriptional start site (TSS) were selected. **b** Venn diagram of LINC02273 target selection. **c** KEGG analysis of the 182 candidate genes. **d** LINC02273 DNA binding were confirmed by ChIRP-qPCR with indicated probes. LacZ probes were used as negative control. **e** RT-qPCR analysis of candidate genes in WT and LINC02273 KO cells. log2 fold change were shown. n = 3, biological replicates. **f** RT-qPCR analysis of AGR2 expression in WT and LINC02273 KO cells. *n* = 3, biological replicates. **g** Immunoblotting of AGR2 after LINC02273 KO. **h** Luciferase reporter assay with LINC02273 overexpression. Left, schematic view of LINC02273 binding site on AGR2 TSS. Truncations were ligated to pGL3-basic vector. Right, luciferase assay was performed with co-transfection of pcDNA3.1-LINC02273 and full length or truncated reporter vectors. **i** Representative images of ChIRP-seq and ChIP-seq results with indicated probes or antibodies on AGR2 genomic region. The peak range of each track were indicated in the square brackets. AGR2-peak represents the DNA region that cloned and used in (**h**). **j** Transwell migration and invasion assay with ectopically overexpressed AGR2. Scale bars: 100 μm. n = 3, biological replicates. **k** Representative images of BLI, 8 weeks after tail vein injection of indicated cells. Photon flux was calculated and compared (*n* = 6, Mann-Whitney test). **l** H&E staining of lung metastasis. 40× images of the red rectangular regions were shown. Scale bars: black, 2 mm; pink, 100 μm. For (**d**-**f**), (**h**), and (**j**-**k**), means ± s.e.m. are shown, and *P* values were determined by independent sample t-tests. *P < 0.05, **P < 0.01, ***P < 0.001, *ns* not significant

To gain insights into the molecular mechanisms underlying the increased metastasis by LINC02273 in breast cancer, we performed RNA sequencing in wild type and two LINC02273 knockout MDA-MB-231 cells (Fig. 5a) and identified 5741 Genes with significant expression change (Fold change > 1.5) in both knockout cell lines. Given the fact that LINC02273 was mainly located in the nucleus, we performed ChIRP-seq to identify the potential DNA binding loci of LINC02273 in MDA-MB-231 cells (Fig. 5a; Additional file 1: Figure S5A-B). Probes were divided into odd or even pool according to the order from 5′ to 3′ targeting LINC02273. Peaks detected in both of the groups were subjected to analysis. We identified 5229 genomic regions that LINC02273 could possibly bind (Fig. 5b). Merged with RNA-seq data, 182 candidate genes were identified as potential direct downstream targets of LINC02273 (Fig. 5b). Gene ontology analysis showed that these genes were significantly associated with cancer development and metastasis, such as PI3K-Akt signaling pathway, MAPK signaling pathway, and focal adhesion (Fig. 5c), supporting the role of LINC02273 in cell proliferation and metastasis processes. Genes with LINC02273 binding enrichment over 10-fold were further verified by ChIRP-qPCR and RT-qPCR. All eight tested genes (CHRNA7, ERP27, GABRG1, FOXN4, ZNF831, AGR2, EBF1 and GUCY2C) were significantly enriched of LINC02273-binding compared to lacZ control (Fig. 5d). Several of these 8 genes, such as AGR2, EBF1, GUCY2C, ZNF831 and CHRNA7, have been shown to regulate cancer progression and may serve as biomarkers [6, 29, 40, 43, 48]. Furthermore, the level change of these genes in LINC02273-KO cells determined by RNA-seq were validated by RT-qPCR (Fig. 5e).

Interestingly, we found LINC02273 bound to the promoter region of AGR2 and GUCY2C, indicating a potential regulatory role of LINC02273 on transcription of these genes (Fig. 5i; Additional file 1: Figure S5C). Previous studies have demonstrated the oncogenic role of AGR2 in several solid tumors including breast cancer [7, 42, 44, 46]. Increased AGR2 expression was associated with decreased recurrence-free survival [1]. Consistent with RNA-seq data, AGR2 levels was significantly decreased in LINC02273-depleted cells, determined by qPCR and immunoblotting (Fig. 5f, g; Additional file 1: Figure S5D). ChIRP-seq analysis revealed that LIN02273 bound to 1756–2150 bp (chr7:16,806,871-16,807,264) upstream of the transcription start site (TSS) of AGR2 (Fig. 5i). To confirm the ChIRP-seq result, luciferase reporters were constructed by fusing the enriched peak region in AGR2 promoter to the luciferase gene. The ectopic expression of LINC02273 increased the luciferase reporter activity compared to the control (Additional file 1: Figure S5E). Further analyses narrowed down the minimal binding site of LINC02273 to Fragment 2 (F2) within AGR2 promoter region (Fig. 5h). Histone modifications plays a vital role in epigenetic regulation of gene transcription. UCSC genome browser showed the AGR2 promoter region was enriched with H3K27ac and H3K4me3 marker (Additional file 1: Figure S5F). ChIP-seq and ChIP-qPCR were performed to investigate whether LINC02273 could regulate AGR2 transcription through altering histone modifications. As expected, H3K27ac and H3K4me3, two histone markers for transcription activation, were decreased after LINC02273 knockout compared to that in wild type cells (Fig. 5i, Additional file 1: Figure S5G). In conclusion, LINC02273 could bind to AGR2 promoter region and promoted the transcriptional activation of AGR2, at least in part, through enhancing chromatin H3K4me3 and H3K27ac modification.

Previous studies have shown that increased AGR2 expression is associated with cancer progression and metastasis in breast cancer [9, 51]. As expected, the mobility and invasiveness breast cancer cells were substantially increased in AGR2-overexpressed MDA-MB-231 and BT549 cells (Fig. 5j; Additional file 1: Figure S6A). Overexpression of AGR2 could rescue the decreased migration ability of LM2 cells due to LINC02273-depletion (Additional file 1: Figure S6B-C). In addition, AGR2 upregulation enhanced lung metastasis of LM2 cells and rescued the metastasis phenotype impaired by LINC02273 knockout in vivo (Fig. 5k-l).

### hnRNPL-LINC02273 complex promotes cancer metastasis through upregulating AGR2

**Fig. 6:**
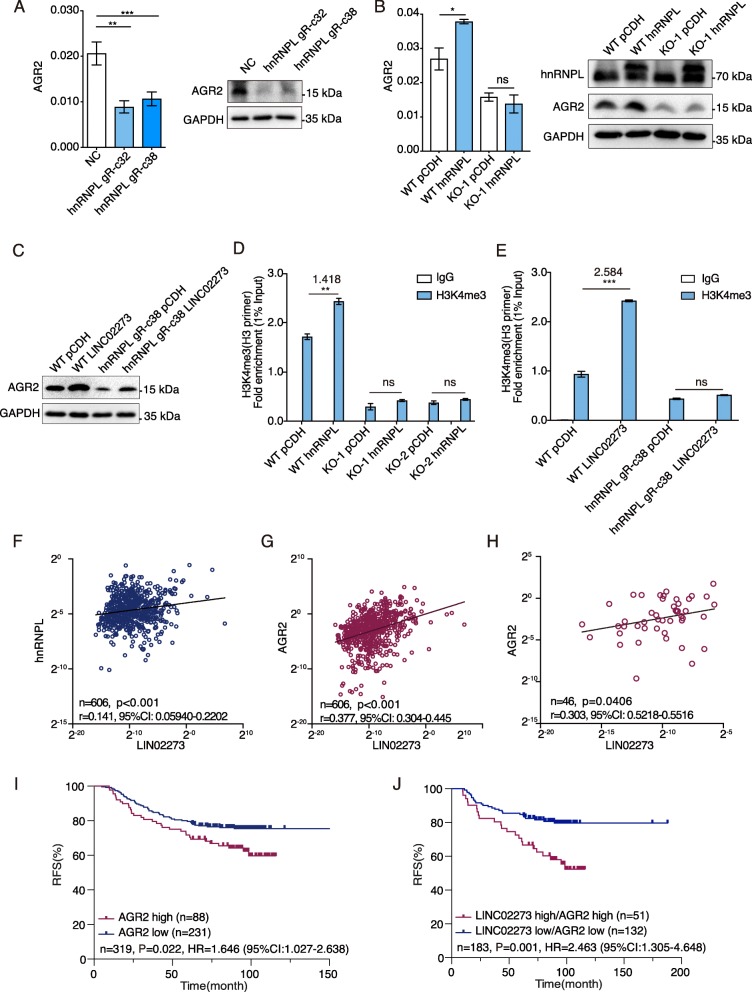
hnRNPL-LINC02273 promotes breast cancer metastasis through AGR2. **a** Relative mRNA and protein level of AGR2 after hnRNPL knockdown in MDA-MB-231 cells (two clones named gRNA32 and gRNA38) assayed by RT-qPCR and immunoblotting. Normalized to GAPDH. n = 3, biological replicates. **b** AGR2 mRNA and protein expression level in WT and LINC02273 KO MDA-MB-231 cells with or without hnRNPL overexpression assayed by RT-qPCR (normalized to GAPDH) and immunoblotting. *n* = 3, biological replicates. **c** AGR2 protein expression level in WT and hnRNPL knockdown MDA-MB-231 cells with or without LINC02273 overexpression by immunoblotting. **d** ChIP-qPCR was performed in WT and LINC02273 knockout MDA-MB-231 cells overexpressed with hnRNPL or pCDH empty vector with indicated antibodies. Normalized to 1% input. *n* = 3, biological replicates. **e** ChIP-qPCR was performed in WT and hnRNPL knockdown (hnRNPL-gRNA38) MDA-MB-231 cells overexpressed with LINC02273 or pCDH empty vector with indicated antibodies. Normalized to 1% input. *n* = 3, biological replicates. **f** Correlation analysis of the gene expression profile between hnRNPL and LINC02273. **g** Correlation analysis of the gene expression profile between AGR2 and LINC02273. For (**f**-**g**), RNA expression data from 606-patient cohort were used (Spearman r). **h** Correlation analysis between AGR2 and LINC02273 in 46 LN samples (Spearman r). **i** Kaplan-Meier RFS analysis of AGR2 expression in 319-patient cohort (log rank test). **j** Kaplan-Meier RFS analysis of AGR2 and LINC02273 expression in 319-patient cohort. Cutoff for LINC02273 was same to Fig. 1f (log rank test). For (**a**-**e**) means ± s.e.m. are shown, and *P* values were determined by independent sample t-tests. *P < 0.05, **P < 0.01, ***P < 0.001, *ns* not significant

As LINC02273 was stabilized by hnRNPL, we wondered whether hnRNPL could also regulate AGR2 through LINC02273. We found that hnRNPL knockdown decreased AGR2 expression significantly, supporting that AGR2 is regulated by hnRNPL (Fig. 6a; Additional file 1: Figure S6D). As hnRNPL bound with LINC02273 to form a complex, we performed ChIP-qPCR to investigate whether hnRNPL was co-localized at AGR2 promoter region with LINC02273. We found that hnRNPL was enriched in the same region of AGR2 promoter (Additional file 1: Figure S6E), likely along with LINC02273. To investigate whether LINC02273 was essential for hnRNPL to regulate AGR2, LINC02273 knocked-out cells were ectopically overexpressed LINC02273 or hnRNPL. We found that ectopically overexpressed LINC02273 could restore decreased AGR2 expression in LINC02273 knock-out cells (Additional file 1: Figure S6F). Although, hnRNPL overexpression in wild-type MDA-MB-231 cells upregulated AGR2, it failed to increase AGR2 mRNA level in LINC02273 knock-out cells (Fig. 6b). In addition, AGR2 transcription were increased after LINC02273 overexpression, even in cells with reduced hnRNPL expression (Fig. 6c, Additional file 1: Figure S6G). Nevertheless, hnRNPL may be required for LINC02273 to fully activate AGR2 transcription, as the level of AGR2 upregulation by LINC02273 was decreased compared to that in WT cells. These data suggested that hnRNPL and LINC02273 collaboratively upregulate the transcription of AGR2 in breast cancer cells.

We further found that overexpression hnRNPL significantly increased H3K4me3 and H3K27ac modification in AGR2 promoter region which was decreased by hnRNPL downregulation (Fig. 6d-e; Additional file 1: Figure S6H-I). Moreover, hnRNPL failed to increase H3K4me3 or H3K27ac modification in LINC02273 knock-out cells (Fig. 6d; Additional file 1: Figure S7A-B), which suggested that the epigenetic regulation by hnRNPL at AGR2 promoter was dependent on LINC02273. Interestingly, the increase of H3K4me3 and H3K27ac levels by LINC02273 overexpression was not observed in cells depleted of hnRNPL (Fig. 6e; Additional file 1: Figure S7C-D). These data indicated that LINC02273 and hnRNPL could enhance AGR2 expression by cooperatively upregulating H3K27ac and H3K4me3 level in AGR2 promoter region, leading to increased breast cancer metastasis.

To further investigate the pathological significance of hnRNPL-LINC02273 axis in breast cancer progression, we analyzed the expression levels of hnRNPL, LINC02273 and AGR2 in primary samples from 606 invasive breast cancer patients (Additional file 1: Figure S7E). LINC02273 was positively correlated with AGR2 and hnRNPL in these patients. Meanwhile, hnRNPL was also positively correlated with AGR2 (Fig. 6f-g). Furthermore, the positive correlation between LINC02273 and AGR2 was also observed in the available metastatic LN samples from the same patient cohort (Fig. 6h). In addition, survival analysis showed that high expression of AGR2 was associated with a poor RFS (*P* = 0.022, HR = 1.646, Fig. 6i, baseline information in Additional file 1: Table S7). The survival benefit (RFS) in patients with low expression of LINC02273 and AGR2 was even more prominent compared to those patients with high expressions of both genes (*P* = 0.001, HR = 2.463) (Fig. 6j). Multivariant COX regression analysis also indicated that high LINC02273/high AGR2 expression is an independent prognostic factor in breast cancer (HR = 2.528, *P* = 0.001, Additional file 1: Table S9). Taken together, these data strongly support that high LINC02273 expression correlates with poor RFS in breast cancer, and downstream increase of AGR2 expression in the hnRNPL-LINC02273 axis may promotes breast cancer metastasis.

### Targeting LINC02273 with ASO showed potential in mitigating breast cancer metastasis

**Fig. 7:**
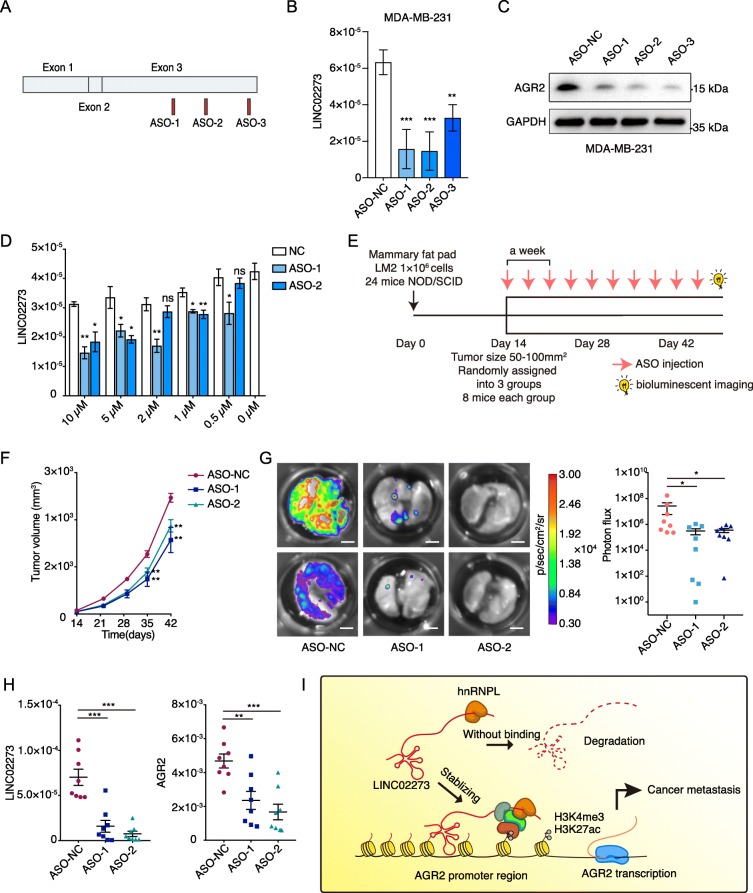
Potential therapeutic role of LINC02273 in breast cancer. **a** Schematic view of ASO targets on LINC02273 mRNA. **b** Relative expression of LINC02273 48 h after ASO transfection were determined by RT-qPCR (normalized to GAPDH). *n* = 3, biological replicates. **c** Immunoblotting analysis of AGR2 expression after ASO transfection in MDA-MB-231 cells. GAPDH were served as loading control. *n* = 3 independent experiments. **d** RT-qPCR showed LINC02273 expression after ASO delivery without transfect reagents. ASO were added into LM2 wild type cells at the concentration as indicated. After 48 h, RNA was extracted, and RT-qPCR was performed. Normalized to GAPDH, *n* = 3, biological replicates. **e** Schematic view of ASO treatment xenograft model. LM2 WT cells were injected into mammary fat pad. Mice were randomized into 3 groups 14 days later. ASO were diluted in PBS and delivered through tail vein twice a week with the concentration of 10 nmol foreach mouse. ASO NC targeted no known sequence with the same length of ASO-1 and ASO-2 were used as negative control. Tumor sizes were measured during treatment. Mice were sacrificed on the day 45. BLI was performed ex vivo. **f** Tumor volumes were measured in the NC and ASO groups in the xenograft mouse model (*n* = 8, independent t-test). **g** Representative images of BLI of lungs and statistical analysis of photon flux. Scale bar: 4 mm. Statistical analysis of total photon flux is on the right (*n* = 8, Mann-Whitney test). **h** LINC02273 and AGR2 expression in xenograft tumor were determined by RT-qPCR (n = 8, Mann-Whitney test). **i** Schematic diagram of hnRNPL-LINC02273-AGR2 axis regulating breast cancer metastasis. For (**b**), (**d**), and (**f**-**g**)**,** means ± s.e.m. are shown. For (**b**) and (**d**), *P* values were determined by independent sample t-tests. *P < 0.05, **P < 0.01, ***P < 0.001

Recently, antisense oligonucleotide (ASO) drugs has gained increasing attention owing to their ability targeting diverse RNAs, which has been validated both in vitro and in vivo [15, 37]. To explore the possibility whether LINC02273 could be interfered by ASO, three ASOs specifically targeting LINC02273 and one negative control targeting no known sequence in human genome were designed for the further study (Fig. 7a). LINC02273 mRNA expression was significantly inhibited by all three ASOs in MDA-MB-231, LM2 and BT549 cells (Fig. 7b; Additional file 1: Figure S8A). Cell proliferation, migration and invasion abilities were also impaired after LINC02273 interference by ASOs, compared to ASO-NC group (Additional file 1: Figure S8B-C). Accordingly, AGR2 expression was dramatically decreased when LINC02273 was suppressed (Fig. 7c; Additional file 1: Figure S8D). We further developed LINC02273-targeting ASO-1/ASO-2 and control ASO-NC with modification optimized for in vivo study. Free uptake assay showed dose-dependent inhibition of LINC02273 in LM2 cells by ASO-1 and ASO-2 compared to NC (Fig. 7d). Orthotopic xenograft tumor model was used to determine the therapeutic efficacy of LINC02273 ASO treatment. Wild type LM2 cells were inoculated into mammary fat pads of NOD/SCID mice (Fig. 7e). After 2 weeks, mice were randomly assigned into three groups (ASO-1, ASO-2 and ASO-NC) and given respective ASO treatment by tail vein injection twice a week. Compared to ASO-NC, tumor growth was significantly decreased in ASO-1 and ASO-2 group (Fig. 7f). More prominently, significant reduction of lung metastasis was observed by bioluminescent imaging, which was further confirmed by H&E staining (Fig. 7g; Additional file 1: Figure S8E). Moreover, we confirmed that the expression of LINC02273 and AGR2 mRNA levels were decreased in tumors tissues treated with ASO-1 and ASO-2, compared to ASO-NC group (Fig. 7h). Collectively, these data demonstrated that LINC02273 played critical role in promoting breast cancer metastasis and targeting LINC02273 with ASO might serve as an effective therapeutic approach to mitigate breast metastasis.

## Discussion

Through profiling transcriptome of metastatic LNs and matched primary cancer tissues, we identified LINC02273 as a lncRNA significantly enriched in LN metastatic lesions, compared to the primary tumors. We further showed that downregulation of LINC02273 impaired the migration and invasion ability of breast cancer cells while its overexpression significantly promoted breast cancer cell mobility. Moreover, LINC02273 knock-down remarkably reduced lung metastasis in breast cancer xenograft models. In breast cancer patients, increased LINC02273 was associated with high burden of LN metastasis. Patients with high LINC02273 level had shorter RFS and worse clinical outcome than those with low LINC02273 expression. Therefore, LINC02273 could serve as an independent prognostic factor in breast cancer.

Recent studies highlighted the critical roles that RNA binding proteins (RBP) may play in modulating cancer initiation and progression through binding to RNAs and regulating their processing, stability, localization, modification or translation [3, 10]. Our mechanistic studies revealed that LINC02273 was stabilized by interaction with hnRNPL. hnRNPL is a RNA binding protein which predominantly binds to the consensus CA repeat motif within RNA [49]. We confirmed that hnRNPL bound to the CA repeats of LINC02273 with its RRM1&2 motif, which is essential for hnRNPL-dependent stabilization of LINC02273. Previous reports indicated that hnRNPL played critical roles in cancer progression [8, 17, 24]. For instance, hnRNPL could promote lymphatic metastasis of bladder cancer through binding with LNMAT1 [4]. hnRNPL was also involved in prostate cancer progression through regulating the alternative splicing of a set of RNAs, including those encoding androgen receptor, the key lineage-specific oncogene of prostate cancer [8]. Here we demonstrated that hnRNPL-promoted breast cancer metastasis was dependent on, at least in part, stabilizing LINC02273. Increased level of hnRNPL was found in metastatic LNs compared to that in matched primary tumors. These results revealed a novel mechanism by which hnRNPL promoted breast cancer progression via its RNA-binding activity.

The molecular mechanisms by which lncRNAs exert their biological functions are diverse and complex. Accumulating evidence suggests that lncRNAs can affect neighboring intra-chromosomal genes *in cis* or genes on different chromosomes *in trans.* Transcriptional regulation by lncRNAs may depend on their roles in modulating signaling transduction, regulating transcription factor recruitment and histone modification, as well as serving as a scaffold for assembly of multiple regulatory molecules at single locus. Since LINC02273 mainly localized in the nucleus, RNA-seq and ChIRP-seq were carried out to identify genes regulated by LINC02273 and respective DNA loci that LINC02273 bound. A number of LINC02273-regulated gene, such as CHRNA7, ERP27, GABRG1, FOXN4, ZNF831, AGR2, EBF1 and GUCY2C, have been reported to be involved in cancer progression and metastasis. CHRNA7 promoted pancreatic and lung cancer metastasis [34, 39], while EBF1 could inhibit colorectal cancer cell proliferation and induce cell apoptosis through the p53/p21 pathway [40]. As a member of protein disulfide isomerase family, AGR2 is involved in maturation of proteins in endoplasmic reticulum and participates in maintaining endoplasmic reticulum homeostasis. Recently studies have revealed varying oncogenic roles of AGR2 in cancer biology. It has been reported that AGR2 could regulate epithelial-mesenchymal transition (EMT) in cancer development [5]. Overexpression of AGR2 exhibited enhanced cancer cell adhesion to a plastic substratum and extracellular AGR2 enhanced the rate of cancer cell attachment [25]. In breast cancer, increased AGR2 had an unfavorable impact on survival [45]. Upregulation of AGR2 could decrease cancer cell sensitivity to fulvestrant and epirubicin [22, 23]. Furthermore, AGR2 inhibits p53 activation after DNA damage [12]. Although the expression of AGR2 has been reported to be regulated by ERK, ER and HIF signaling pathways as well as by its 3’UTR [5, 13, 23, 31], the molecular mechanisms controlling AGR2 transcription remains poorly understood. In our study, we found a novel mechanism that oncogene AGR2 could be modulated by LINC02273. LINC02273 binds to AGR2 promoter along with hnRNPL which changes its epigenetic landscape. Enrichment of LINC02273-hnRNPL complex at AGR2 promoter increased local H3K4me3 and H3K27ac levels, thereby promoting AGR2 transcription. It is plausible that hnRNPL-bound LINC02273 serves as a scaffold and a guide RNA to recruit additional histone writer proteins and/or transcriptional coactivators to AGR2 promoter region, which enhances AGR2 transcription. The LINC02273-recuited histone writers responsible for increased H3K4me3 and H3K27ac levels and other potential transcriptional activators warrants further investigation.

In addition, several studies have reported the role of hnRNPL in regulating gene transcription. For instance, hnRNPL plays an important role in gene regulation in epithelial cells [8]. Recent studies have also demonstrated that hnRNPL might be involved in transcriptional regulation through histone modifications [21]. We found hnRNPL enhanced AGR2 transcription in breast cancer cells. Our ChIP analysis revealed that hnRNPL was enriched in the same AGR2 promoter region where LINC02273 bound. Knock-down of hnRNPL decreased the histone H3K4me3 and H3K27ac around AGR2 promoter region. Furthermore, overexpressing hnRNPL failed to rescue the decreased H3K4me3 and H3K27ac at AGR2 promoter region, and AGR2 transcription in LINC02273-knockout cells, indicating that the transactivating effect of hnRNPL on AGR2 promoter was dependent on LINC02273. In reciprocal, increased LINC02273 barely restored the decreased H3K4me3 and H3K27ac modifications in hnRNPL-depleted cells, supporting an essential role of hnRNPL-LINC02273 complex in enhancing transactivating histone modification at AGR2 promoter region. Nevertheless, we noticed that AGR2 transcription were increased by LINC02273 overexpression, although at a reduced level, in hnRNPL-depleted cells. Therefore, beside hnRNPL, LINC02273 may bind additional transcription regulators to enhance AGR2 expression, which remain to be identified.

Consistent with our in vitro findings, a positive correlation between LINC02273 and AGR2 was confirmed in both primary tumors and metastatic LNs from breast cancer patients. In addition, shorter RFS was significantly associated with high expression of both AGR2 and LINC02273, suggesting combined expression levels of AGR2 and LINC02273 may serve as promising biomarkers to determine prognosis in breast cancer.

Overall, hnRNPL mRNA expression was relative stable in breast cancer samples (RPKM mean = 138, mean fold change = 4.7, coefficient of variation = 20.8% )[28]. Indeed, we also found hnRNPL expression displayed relative low variance in our cohort, although increased expression of hnRNPL in metastatic LNs, compared to the matched primary tumors, was observed. On the contrary, we found LINC02273 was overexpressed in breast tumor samples with high variance. In addition, knockout or knockdown of LINC02273 could significantly curb cell migration and invasion ability, which indicates its potential as a promising therapeutic target for antagonizing breast cancer metastasis. Recently, antisense oligos (ASO) and locked nucleic acid (LNA) has been used to target mRNAs in vivo and several ASOs have been investigated in the clinical trials [ClinicalTrials Identifier: NCT01839604, NCT02423590 ][2, 11, 15, 37, 50]. We explored whether LINC02273 expression could be inhibited by ASO both in vitro and in vivo*.* Our data indicated that ASO could effectively suppress LINC02273 in cells and xenograft tumors. Moreover, LINC02273-targeting ASOs substantially reduced primary tumor growth and lung metastasis in our xenograft model. The observation strongly supports that suppressing LINC02273 with specific ASOs may serve as an effective therapeutic approach to mitigate LINC02273-promoted breast cancer progression.

## Conclusions

Our study revealed that LINC02273, as a novel metastasis-associated onco-lncRNA, promoted tumor invasion and correlated with poor prognosis in breast cancer. Mechanistically, LINC02273 was stabilized by binding of hnRNPL to the CA-repeats within its S3–2 region. The hnRNPL-LINC02273 RNP complex could be recruited to the promoter region of AGR2 where it enhanced AGR2 transcription by increasing local H3K4me3 and H3K27ac modification (Fig. 7i). LINC02273-hnRNPL-AGR2 axis may serve as prognostic biomarkers and promising therapeutic targets for mitigating breast cancer metastasis and progression.

## Additional file

**Additional file 1. Figure S1**. LINC02273 correlates with poor prognosis in breast cancer. **Figure S2**. qPCR analysis of LINC02273 expression and genes closed to LINC02273 gene locus in LINC02273 KO cells. **Figure S3**. LINC02273 promotes breast cancer proliferation and metastasis but not cancer stemness. **Figure S4**. LINC02273 interacts with hnRNPL. **Figure S5**. LINC02273 transcriptionally activates AGR2 expression which was revealed by ChIRP. **Figure S6**. hnRNPL-LINC02273 complex promotes cancer metastasis through upregulating AGR2. **Figure S7**. Epigenetic regulation of modification at AGR2 promoter by hnRNPL was dependent on LINC02273. **Figure S8**. Targeting LINC02273 with ASO showed potential in mitigating breast cancer metastasis. **Table S1**. Patient characteristics of RNA microarray. **Table S2**. CPAT prediction of LINC02273. **Table S3**. CPC prediction of LINC02273. **Table S4**. Baseline clinicopathological characteristics of patients according to LINC02273 expression. **Table S5**. Univariant COX regression analyses of RFS in BC patients. **Table S6**. Multivariant COX regression analyses of RFS in BC patients. **Table S7**. LINC02273 knockout neckties. **Table S8**. MS result of LINC02273 RNA pull-down. **Table S9**. Baseline clinicopathological characteristics of patients according to AGR2 expression. **Table S10**. Multivariant COX regression analyses of RFS in BC patients. **Table S11**. Probes used in this study. **Table S12**. Primers used in this study. **Table S13**. shRNA, siRNA, CRISPR, and ASOs.

## Data Availability

Datasets used and/or analyzed during the current study are available from the corresponding author on reasonable request.

## Abbreviations

AGR2: Anterior gradient protein 2
ChIP-seq: Chromatin immunoprecipitation
ChIRP-seq: Chromatin isolation by RNA purification followed by sequencing
CPAT: Coding Potential Assessment Tool
CPC: Coding Potential Calculator
H3K27ac: Acetylation at the 27th lysine residue of the histone H3 protein
H3K4me3: Tri-methylated at the 4th lysine residue of the histone H3 protein
hnRNPL: heterogeneous nuclear ribonucleoprotein L
HR: Hazard ratio
KO: Knock out
LN: Lymph node
lncRNA: long noncoding RNA
NC: Negative control
RACE: Rapid amplification of cDNA ends
RIP: RNA binding protein immunoprecipitation
RT-qPCR: reverse transcriptase-quantitative real time PCR
WT: Wild type
