# Supplementary material for: LINC02273 drives breast cancer metastasis by epigenetically increasing AGR2 transcription
Source: Mol Cancer. 2019 Dec 19;18:187. doi: 10.1186/s12943-019-1115-y (PMC6921600; doi:10.1186/s12943-019-1115-y)
Supplement: Supplementary file 1 — Additional file 1. Figure S1. LINC02273 correlates with poor prognosis in breast cancer. Figure S2. qPCR analysis of LINC02273 expression and genes closed to LINC02273 gene locus in LINC02273 KO cells. Figure S3. LINC02273 promotes breast cancer proliferation and metastasis but not cancer stemness. Figure S4. LINC02273 interacts with hnRNPL. Figure S5. LINC02273 transcriptionally activates AGR2 expression which was revealed by ChIRP. Figure S6. hnRNPL-LINC02273 complex promotes cancer metastasis through upregulating AGR2. Figure S7. Epigenetic regulation of modification at AGR2 promoter by hnRNPL was dependent on LINC02273. Figure S8. Targeting LINC02273 with ASO showed potential in mitigating breast cancer metastasis. Table S1. Patient characteristics of RNA microarray. Table S2. CPAT prediction of LINC02273. Table S3. CPC prediction of LINC02273. Table S4. Baseline clinicopathological characteristics of patients according to LINC02273 expression. Table S5. Univariant COX regression analyses of RFS in BC patients. Table S6. Multivariant COX regression analyses of RFS in BC patients. Table S7. LINC02273 knockout neckties. Table S8. MS result of LINC02273 RNA pull-down. Table S9. Baseline clinicopathological characteristics of patients according to AGR2 expression. Table S10. Multivariant COX regression analyses of RFS in BC patients. Table S11. Probes used in this study. Table S12. Primers used in this study. Table S13. shRNA, siRNA, CRISPR, and ASOs. [file 12943_2019_1115_MOESM1_ESM.pdf]

Additional file 1

Additional file 1: Figure S1. LINC02273 correlates with poor prognosis in breast cancer.

(A) The flow chart for screening metastasis associated candidate lncRNAs in breast cancer. (B) Representative frozen section images of primary tumor and LN metastatic loci were shown. Red line circled out the tissue dissected for qPCR and microarray. Scare bar 500 μm for 4×, 100 μm for 20×. (C) Microarray result of the mean relative RNA expression of LN metastatic loci compared to corresponding primary tumor in 5 breast cancer patients with statistical significance ($P<0.05$). Mean log2 fold change were shown. (D) Relative mRNA expression of LINC02273 1653nt and 1252nt isoforms in 319 patient cohort (Wilcoxon matched pairs signed rank test, ***P<0.001). Normalized to GAPDH. (E) Relative mRNA expression of 1653nt and 1252nt isoform after 1653nt isoform overexpression measured by RT-qPCR. n=3, biological replicates. Normalized to GAPDH. (F) Relative mRNA expression of LINC2446 and LINC02273 after shRNA knockdown were measured by RT-qPCR. n=3, biological replicates. Normalized to GAPDH. (G) Transwell migration assay after shRNA transfection in MDA-MB-231 cells. Statistical analysis was made according to the cell confluence. n=3, biological replicates. (H) RT-qPCR for LINC02273 expression in human breast cancer with or without lymph node metastasis (Mann-Whitney test, data are shown as median with interquartile range, P=0.048). Normalized to GAPDH. (I)-(K) Kaplan-Meier recurrence-free survival (RFS) analysis of LINC02273 expression in (I) ER positive/PR positive, (J) HER2 positive, (K)Triple negative breast cancer (TNBC), breast cancer patients (log rank test). For (E)-(G), data are means ± s.e.m., *$P < 0.05$, **$P < 0.01$, ***$P < 0.001$ and P values were determined by independent sample t-test.

Additional file 1: Figure S2. qPCR analysis of LINC02273 expression and genes closed to LINC02273 gene locus in LINC02273 KO cells.

(A) Kaplan-Meier metastasis-recurrence-free (MR-free) survival analysis of LINC02273 expression in 351 breast cancer patients from bc-GenExMiner 3.0 database (log rank test). (B) Relative mRNA expression of LINC02273 in breast cancer cell lines were measured by RT-qPCR. n=3, biological replicates. (C) Schematic view of LINC02273 knockout in MDA-MB-231 cells. Up, sanger sequencing after PCR with genomic DNA using primer indicated in the graph. Bottom, sanger sequencing reads of KO-1 and KO-2 MDA-MB-231 cells. (D) Relative expression of genes closed to LINC02273 gene locus, as presented in Fig. 1A, in MDA-MB-231 and LM2. Measured by RT-qPCR. n=3, biological replicates. Normalized to GAPDH. (E) Representative image of genotyping after LINC02273 knockout in MDA-MB-231, BT-549 and LM2 cells by using primer in (B) and subjected to DNA electrophoresis. (F) RT-qPCR showed LINC02273 was ectopically overexpressed in MDA-MB-231 and BT549 cells. n=3, biological replicates. For B, D, and F data are means ± s.e.m. *P* values were determined by independent sample t-tests. *$P<0.05$, **$P < 0.01$, ***$P < 0.001$.

Additional file 1: Figure S3. LINC02273 promotes breast cancer proliferation and metastasis but not cancer stemness.

(A) RT-qPCR showed LINC02273 was knocked out in MDA-MB-231, BT549, and LM2 cells. n=3, biological replicates. (B) Proliferation assay was performed after LINC02273 knockout in MDA-MB-231 and BT549 cells. Cell confluence was measured. Statistical analysis was performed on the last three time points. Results are shown as the means ± s.e.m. from three independent experiments. (C)Transwell migration assay and invasion assay were performed after LINC02273 overexpression in MDA-MB-231, (D) BT549 cells, and (E) LM2 cells. Representative photos and quantitative analysis are shown. Confluence of migrated cells were analyzed by ImageJ. Scale bar, 100 mm. n=3, biological replicates. (F) Mammosphere-forming assay of wild type and LINC02273 knockout cells in MDA-MB-231, LM2, and BT549. Mammospheres over 100 μm were counted under microscope. Representative photos and quantitative analysis are shown. Scare bar 500 μm for 4×, 200 μm for 10 ×. For (A)-(F) data are means ± s.e.m. *P* values were determined by independent sample t-tests. **P*<0.05, ***P* < 0.01, ****P* < 0.001.

Additional file 1: Figure S4. LINC02273 interacts with hnRNPL.

(A) Representative image of gel band analysis after silver staining in Fig. 4A by mass spectrometry. (B) Immunoblotting confirms nuclear-cytoplasmic fractionation efficiency in MDA-MB-231. hnRNPL was enriched in nuclear fraction. Lamin B was used as nuclear protein indicator while GAPDH as cytoplasmic fraction indicator. W, whole lysate; N, nuclear fraction; C, cytoplasmic fraction. (C) RIP efficiency in MDA-MB-231 was determined by immunoblotting. Beads only and IgG served as negative control. (D) Schematic view of the predicted secondary structure of LINC02273 RNA. According to secondary structure, we truncated LINC02273 into 3 segments and annotated as S1, S2, and S3. (E) Knockdown efficiency of hnRNPL in MDA-MB-231 and BT549 cells determined by immunoblotting. GAPDH was used as loading control. (F) RT-qPCR showed LINC02273 expression after ectopically overexpressed ETS1, STAT3, and hnRNPL in MDA-MB-231 cells. pcDNA3.1 empty vector was used as negative control. n=3, biological replicates. (G) Luciferase reporter assay of LINC02273 promoter. LINC02273 promoter region as shown in Fig. S2B was cloned into pGL3 vector. pcDNA3.1 empty vector, pcDNA3.1-STAT3, pcDNA3.1-ETS1, and pcDNA3.1-hnRNPL were co-transfected with pGL3-LINC02273 separately. pcDNA3.1 was served as negative control. Results are normalized to pcDNA3.1. n=3, biological replicates. (H) siRNA was used to knockdown hnRNPL. Left, RT-qPCR was performed and analyzed. Right, immunoblotting showed hnRNPL knockdown. siNC targeted no known sequence served as negative control. n=3, biological replicates. (I) Immunoblotting showed hnRNPL was ectopically overexpressed in MDA-MB-231 and BT549 cells. For (F)-(G), RT-qPCR data are normalized to GAPDH, means ± s.e.m. are shown, and P values were determined by independent sample t-tests. *$P < 0.05$, **$P < 0.01$, ***$P < 0.001$, *ns* not significant.

Additional file 1: Figure S5. LINC02273 transcriptionally activates AGR2 expression which was revealed by ChIRP.

(A) RNA pulled down by ChIRP was purified. RT-qPCR was performed to confirm the efficiency of ChIRP. ACTB, GAPDH, U6 were served as negative control. n=3, biological replicates. (B) Representative image of quality control for ChIRP. After DNA purification, Agilent 2100 was used to control sonification efficiency in ChIRP. LM, low molecular weight; HM, high molecular weight. (C) Representative images of ChIRP-seq results with indicated probes on GUCY2C genomic region. The peak range of each track were indicated in the square brackets. (D) RT-qPCR and immunoblotting showed AGR2 expression after LINC02273 knockout in LM2 cells. Data are normalized to GAPDH. n=3, biological replicates. (E) Luciferase reporter assay with LINC02273 overexpression. LINC02273 binding region of AGR2 promoter (AGR2-peak) was cloned into pGL3 basic and pGL3-enhancer (pGL3-basic with SV40 enhancer). Luciferase assay was performed with co-transfection of pcDNA3.1-LINC02273 and reporter vectors. Relative luciferase activity was calculated normalized to empty vector (EV) of pcDNA3.1. n=3, biological replicates. (F) Schematic view of AGR2 promoter region in UCSC genome browser. Layered H3K4me1, H3K4me3, and H3K27ac was shown. Transcriptional start site was indicated by black arrow. (G) ChIP-qPCR was performed in MDA-MB-231 WT and LINC02273 KO cells with indicated antibody. ACTB were used as negative control. AGR2 primer were designed in AGR2-TSS region named H3 primer. n=3, biological replicates. For (A), (D)-(F), and (G), means ± s.e.m. are shown, and P values were determined by independent sample t-tests. *$P < 0.05$, **$P < 0.01$, ***$P < 0.001$.

Additional file 1: Figure S6. hnRNPL-LINC02273 complex promotes cancer metastasis through upregulating AGR2.

(A) RT-qPCR and immunoblotting showed AGR2 overexpression in MDA-MB-231 and BT549 cells. pCDH empty vector were used as negative control. Normalized to GAPDH, n=3, biological replicates. (B) immunoblotting showed AGR2 were overexpressed in LM2 cell lines as indicated. n=3 independent experiments. (C) Transwell migration assay was performed with cell lines from (B). Representative photos and quantitative analysis are shown. Scale bar, 100 mm. n=3, biological replicates. (D) immunoblotting showed AGR2 expression in hnRNPL knockdown BT549 cell (one clone named gR-C2). WT served as negative control. n=3, biological replicates. (E) ChIP-qPCR showed hnRNPL fold enrichment on AGR2 promoter region that LINC02273 binds. (F) RT-qPCR and immunoblotting showed AGR2 expression in WT and LINC02273 KO cells, and ectopic recovered LINC02273 KO cells. Normalized to GAPDH, n=3, biological replicates. (G) AGR2 mRNA expression level in WT and hnRNPL knockdown MDA-MB-231 cells with or without LINC02273 overexpression by RT-qPCR. Normalized to GAPDH. n=3, biological replicates. (H) ChIP-qPCR showed H3K4me3 fold enrichment on AGR2 promoter region that LINC02273 binds. Normalized to 1% input. n=3, biological replicates. (I) ChIP-qPCR showed H3K27ac fold enrichment on AGR2 promoter region that LINC02273 binds. Normalized to 1% input. n=3, biological replicates. For (A), (C), (E)-(I), means ± s.e.m. are shown, and *P* values were determined by independent sample t-tests. $^{*}P < 0.05$, $^{**}P < 0.01$, $^{***}P < 0.001$.

Additional file 1: Figure S7. Epigenetic regulation of modification at AGR2 promoter by hnRNPL was dependent on LINC02273.

(A) Immunoblotting showed hnRNPL expression after ectopically overexpression in MDA-MB-231 cells. (B) ChIP-qPCR was performed in WT and LINC02273 knockout MDA-MB-231 cells overexpressed with hnRNPL or pCDH empty vector with indicated antibodies. Normalized to 1% input. n=3, biological replicates. (C) RT-qPCR showed LINC02273 expression in WT and hnRNPL knockdown cells with or without LINC02273 overexpression. Normalized to GAPDH, n=3, biological replicates. (D) ChIP-qPCR was performed in WT and hnRNPL knockdown (hnRNPL-gR-c38) MDA-MB-231 cells overexpressed with LINC02273 or pCDH empty vector with indicated antibodies. Normalized to 1% input. n=3, biological replicates. (E) Relative expression of hnRNPL, LINC02273 and AGR2 mRNA was determined by RT-qPCR in 606-patient cohort (data are median with interquartile range). For (B)-(D) means ± s.e.m. are shown, *P* values were determined by independent sample t-tests. *$P < 0.05$, **$P < 0.01$, ***$P < 0.001$, *ns* not significant.

Additional file 1: Figure S8. Targeting LINC02273 with ASO showed potential in mitigating breast cancer metastasis.

(A) Relative expression of LINC02273 48h after ASO transfection were determined by RT-qPCR (normalized to GAPDH). (B) Proliferation assay was performed in MDA-MB-231 and BT549 cells with ASO transfection. Cell confluence was measured. Statistical analysis was performed on 120h (independent t-test). n=3, biological replicates. (C) Transwell migration assay and invasion assay were performed after ASO transfection in MDA-MB-231, LM2, and BT549 cells. Representative photos and quantitative analysis are shown. Scale bar, 100 mm. n=3, biological replicates. (D) Relative expression of AGR2 48h after ASO transfection determined by RT-qPCR (normalized to GAPDH) in MDA-MB-231 and LM2 cells. n=3, biological replicates. (E) Representative images of H&E staining of lung metastasis from mice cohort in Fig. 7E. Two representative images were shown from each group. Scale bars: black, 2 mm; pink, 100 μm. For (A)-(D), means ± s.e.m. are shown, and *P* values were determined by independent sample t-tests. *$P < 0.05$, **$P < 0.01$, ***$P < 0.001$.

Figure S1

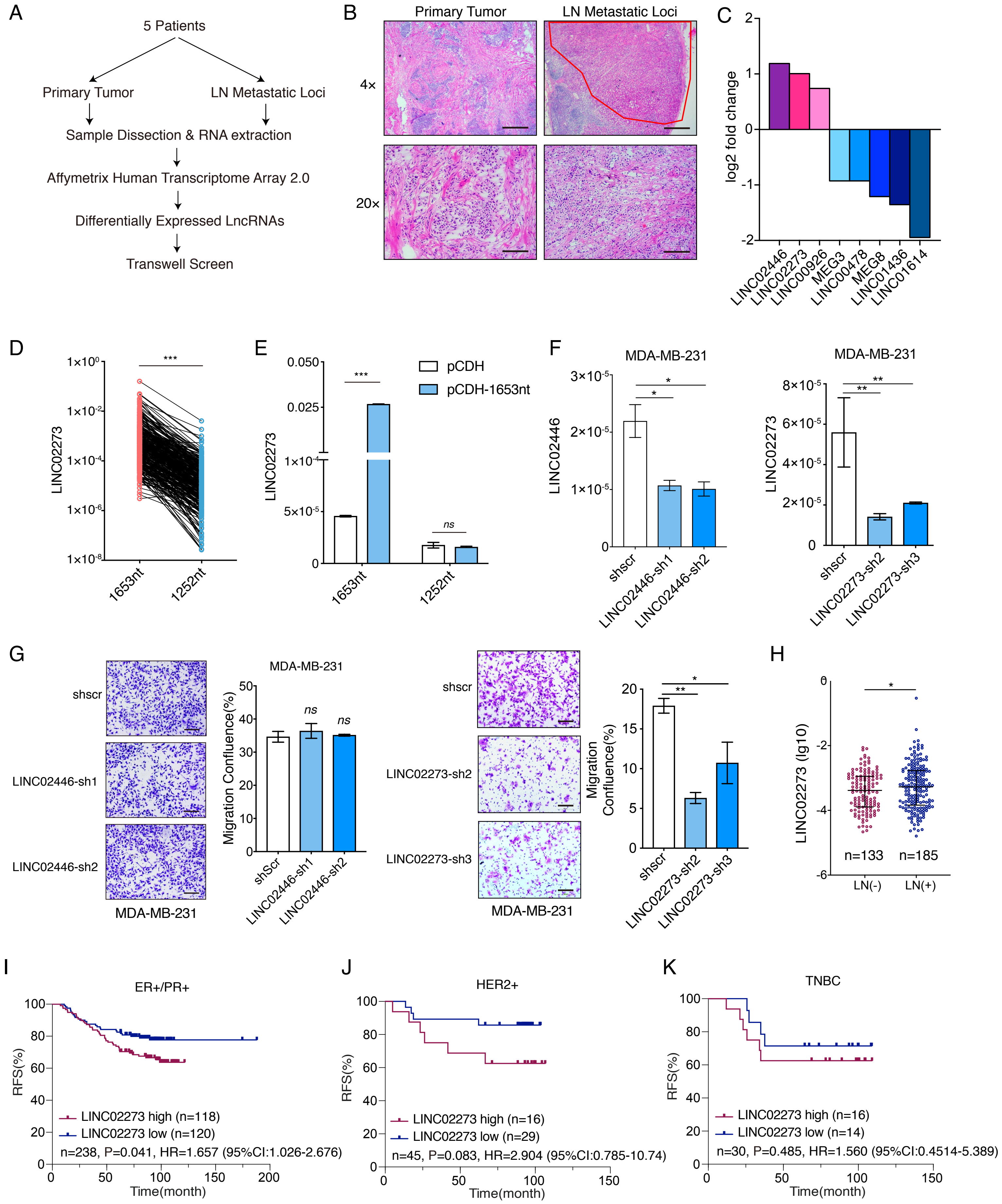
A
5 Patients
Primary Tumor
LN Metastatic Loci
Sample Dissection & RNA extraction
Affymetrix Human Transcriptome Array 2.0
Differentially Expressed LncRNAs
Transwell Screen
B
Primary Tumor
LN Metastatic Loci
4×
20×
C
log2 fold change
LINC02446
LINC02273
LINC00926
MEG3
LINC00478
MEG8
LINC01436
LINC01614
D
LINC02273
1653nt
1252nt
E
pCDH
pCDH-1653nt
LINC02273
1653nt
1252nt
ns
F
MDA-MB-231
LINC02446
shscr
LINC02446-sh1
LINC02446-sh2
MDA-MB-231
LINC02273
shscr
LINC02273-sh2
LINC02273-sh3
G
shscr
LINC02446-sh1
LINC02446-sh2
MDA-MB-231
MDA-MB-231
Migration Confluence(%)
shScr
LINC02446-sh1
LINC02446-sh2
ns
ns
shscr
LINC02273-sh2
LINC02273-sh3
MDA-MB-231
Migration Confluence(%)
shscr
LINC02273-sh2
LINC02273-sh3
H
LINC02273 (lg10)
n=133
n=185
LN(-)
LN(+)
I
ER+/PR+
RFS(%)
LINC02273 high (n=118)
LINC02273 low (n=120)
n=238, P=0.041, HR=1.657 (95%CI:1.026-2.676)
Time(month)
J
HER2+
RFS(%)
LINC02273 high (n=16)
LINC02273 low (n=29)
n=45, P=0.083, HR=2.904 (95%CI:0.785-10.74)
Time(month)
K
TNBC
RFS(%)
LINC02273 high (n=16)
LINC02273 low (n=14)
n=30, P=0.485, HR=1.560 (95%CI:0.4514-5.389)
Time(month)

Figure S2

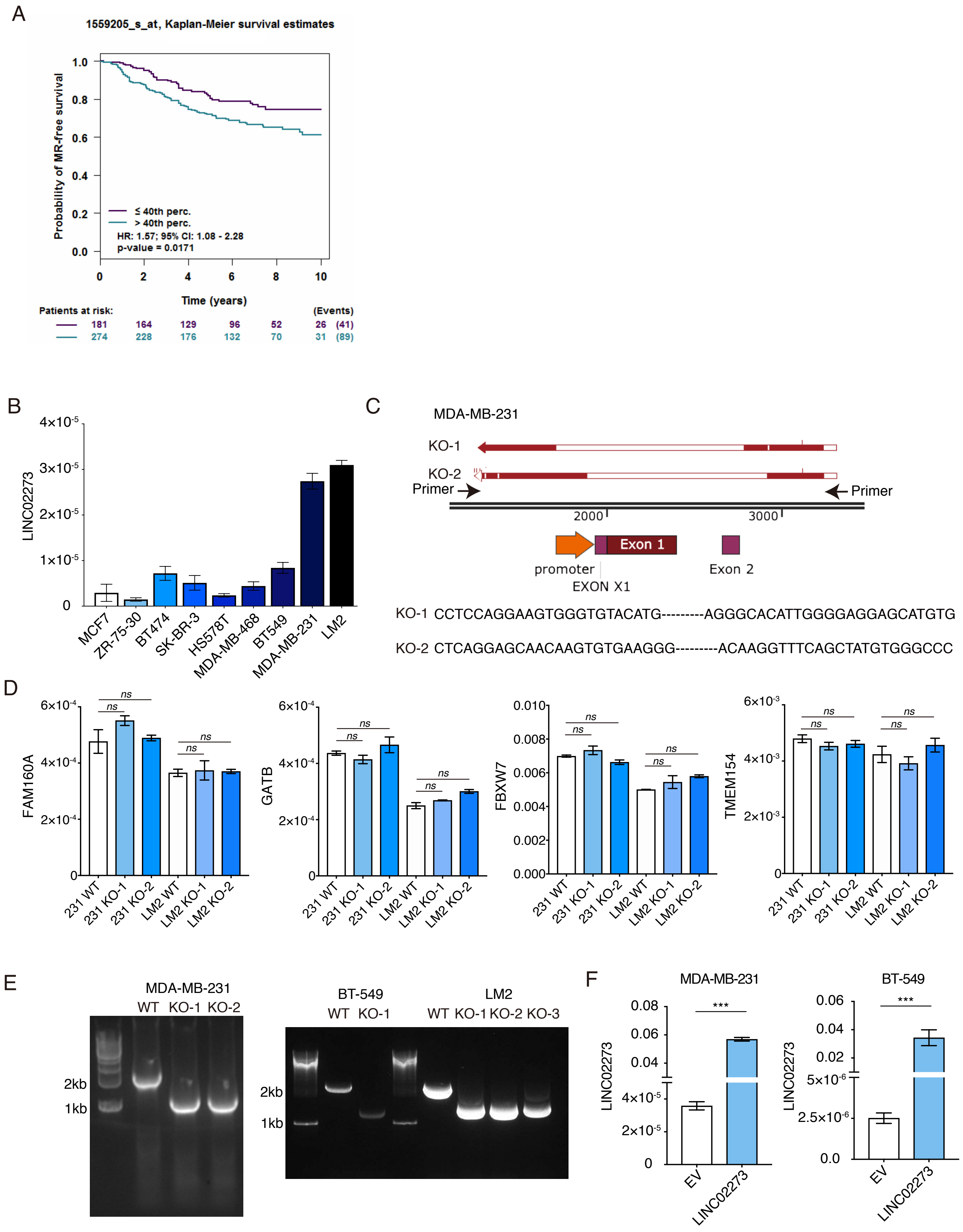
A
1559205_s_at, Kaplan-Meier survival estimates
Probability of MR-free survival
1.0
0.8
0.6
0.4
0.2
0.0
≤ 40th perc.
> 40th perc.
HR: 1.57; 95% CI: 1.08 - 2.28
p-value = 0.0171
0 2 4 6 8 10
Time (years)
Patients at risk:
(Events)
181 164 129 96 52 26 (41)
274 228 176 132 70 31 (89)
B
LINC02273
4x10-5
3x10-5
2x10-5
1x10-5
0
MCF7
ZR-75-30
BT474
SK-BR-3
HS578T
MDA-MB-468
BT549
MDA-MB-231
LM2
C
MDA-MB-231
KO-1
KO-2
Primer
Primer
2000
3000
Exon 1
promoter
Exon 2
EXON X1
KO-1 CCTCCAGGAAGTGGGTGTACATG---------AGGGCACATTGGGGAGGAGCATGTG
KO-2 CTCAGGAGCAACAAGTGTGAAGGG---------ACAAGGTTTCAGCTATGTGGGCCC
D
FAM160A
GATB
FBXW7
TMEM154
ns
6x10-4
4x10-4
2x10-4
0
0.010
0.008
0.006
0.004
0.002
0.000
6x10-3
4x10-3
2x10-3
231 WT
231 KO-1
231 KO-2
LM2 WT
LM2 KO-1
LM2 KO-2
E
MDA-MB-231
WT KO-1 KO-2
2kb
1kb
BT-549
WT KO-1
LM2
WT KO-1 KO-2 KO-3
F
MDA-MB-231
BT-549
LINC02273
***
0.08
0.06
0.04
4x10-5
2x10-5
0
0.06
0.04
0.02
5x10-6
2.5x10-6
0.0
EV
LINC02273

Figure S3

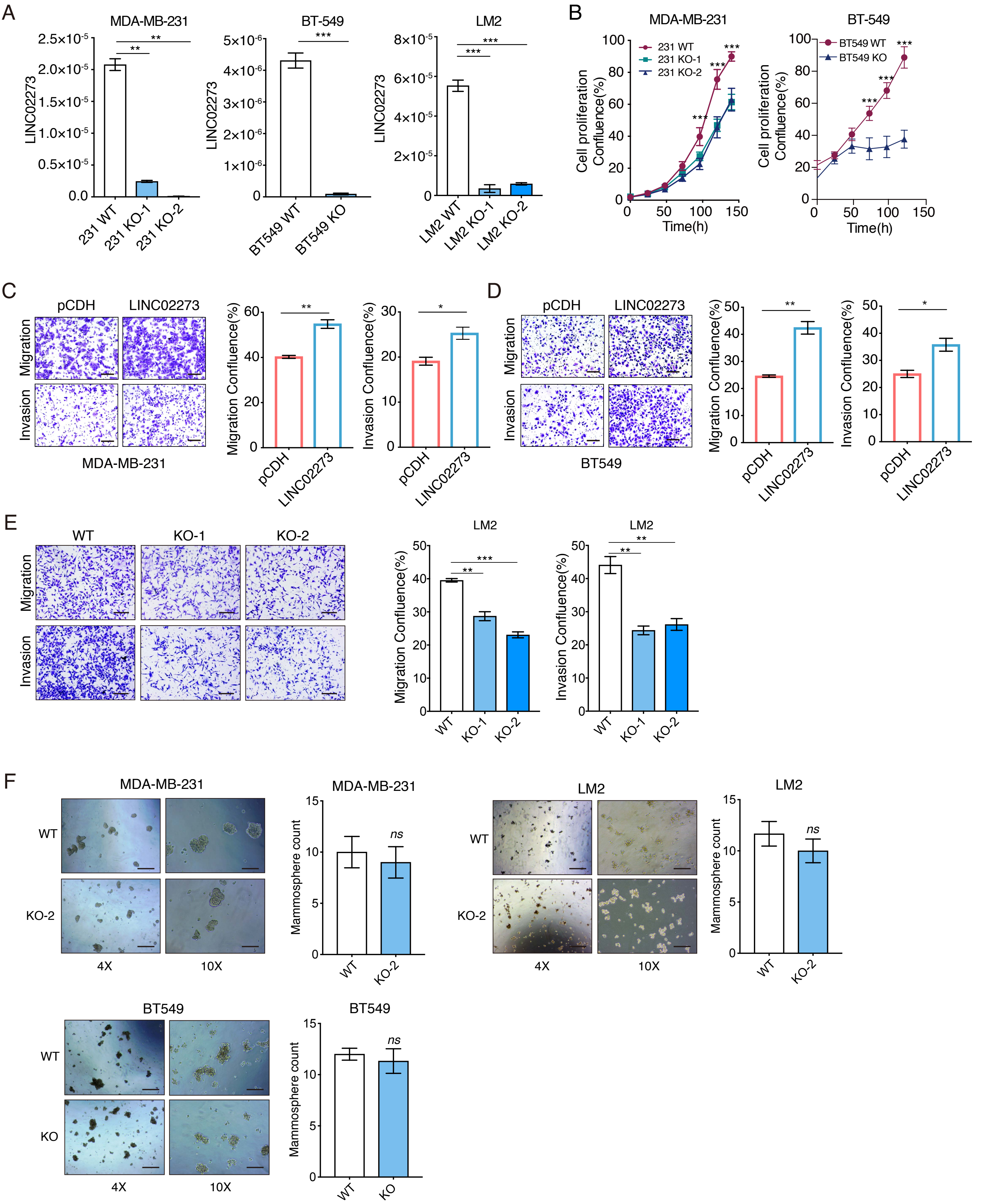

A
MDA-MB-231
BT-549
LM2
LINC02273
231 WT
231 KO-1
231 KO-2
BT549 WT
BT549 KO
LM2 WT
LM2 KO-1
LM2 KO-2
B
MDA-MB-231
BT-549
231 WT
231 KO-1
231 KO-2
BT549 WT
BT549 KO
Cell proliferation Confluence(%)
Time(h)
C
pCDH
LINC02273
Migration
Invasion
MDA-MB-231
Migration Confluence(%)
Invasion Confluence(%)
D
pCDH
LINC02273
Migration
Invasion
BT549
Migration Confluence(%)
Invasion Confluence(%)
E
WT
KO-1
KO-2
Migration
Invasion
LM2
Migration Confluence(%)
Invasion Confluence(%)
F
MDA-MB-231
WT
KO-2
4X
10X
Mammosphere count
ns
LM2
WT
KO-2
4X
10X
Mammosphere count
ns
BT549
WT
KO
4X
10X
Mammosphere count
ns

Figure S4

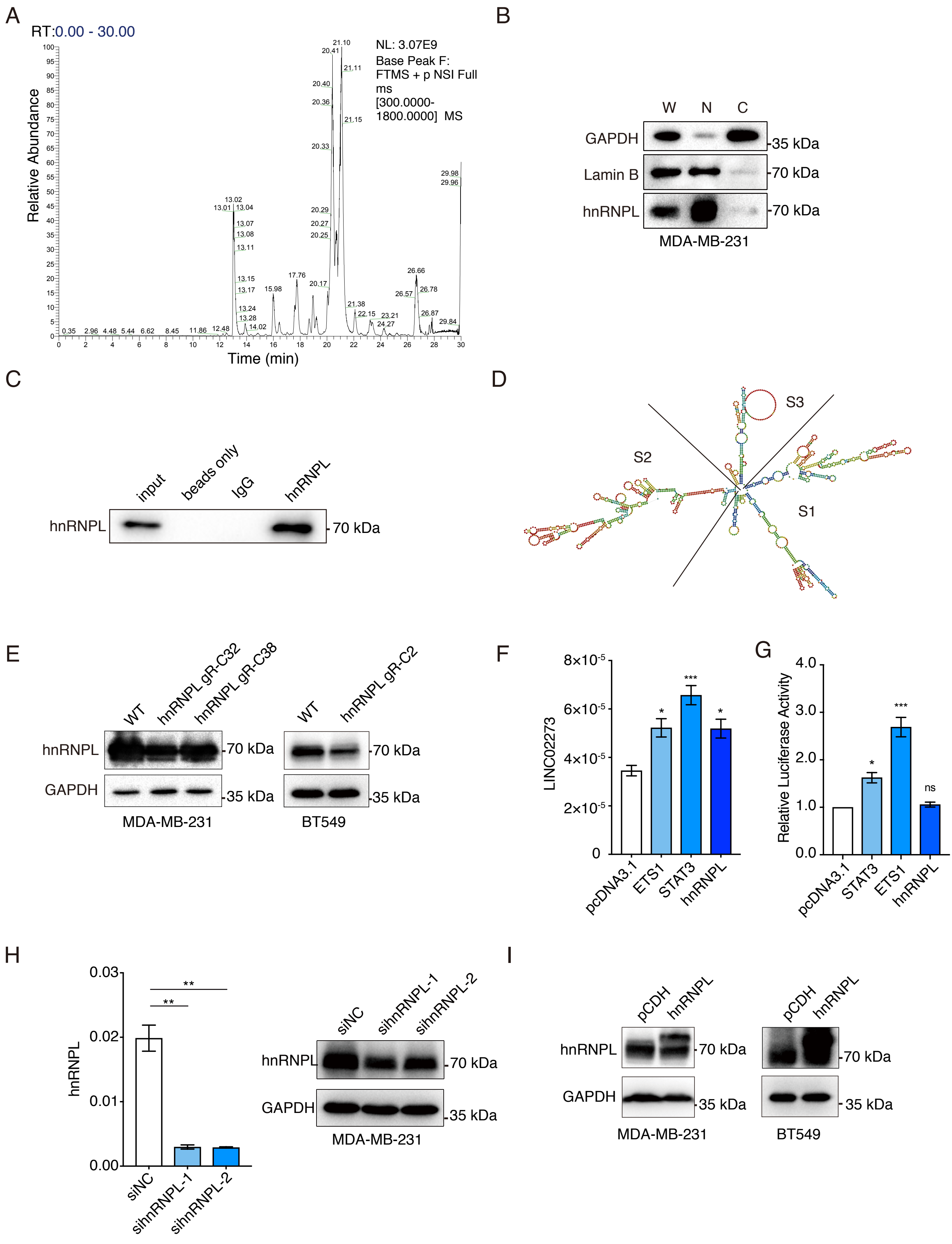
A
RT:0.00 - 30.00
NL: 3.07E9
Base Peak F:
FTMS + p NSI Full
ms
[300.0000-
1800.0000] MS
Relative Abundance
Time (min)
B
W
N
C
GAPDH
Lamin B
hnRNPL
35 kDa
70 kDa
70 kDa
MDA-MB-231
C
input
beads only
IgG
hnRNPL
hnRNPL
70 kDa
D
S1
S2
S3
E
WT
hnRNPL gR-C32
hnRNPL gR-C38
WT
hnRNPL gR-C2
hnRNPL
GAPDH
70 kDa
35 kDa
MDA-MB-231
BT549
F
LINC02273
pcDNA3.1
ETS1
STAT3
hnRNPL
G
Relative Luciferase Activity
pcDNA3.1
STAT3
ETS1
hnRNPL
ns
H
hnRNPL
siNC
sihnRNPL-1
sihnRNPL-2
GAPDH
70 kDa
35 kDa
MDA-MB-231
I
pCDH
hnRNPL
hnRNPL
GAPDH
70 kDa
35 kDa
MDA-MB-231
BT549

Figure S5

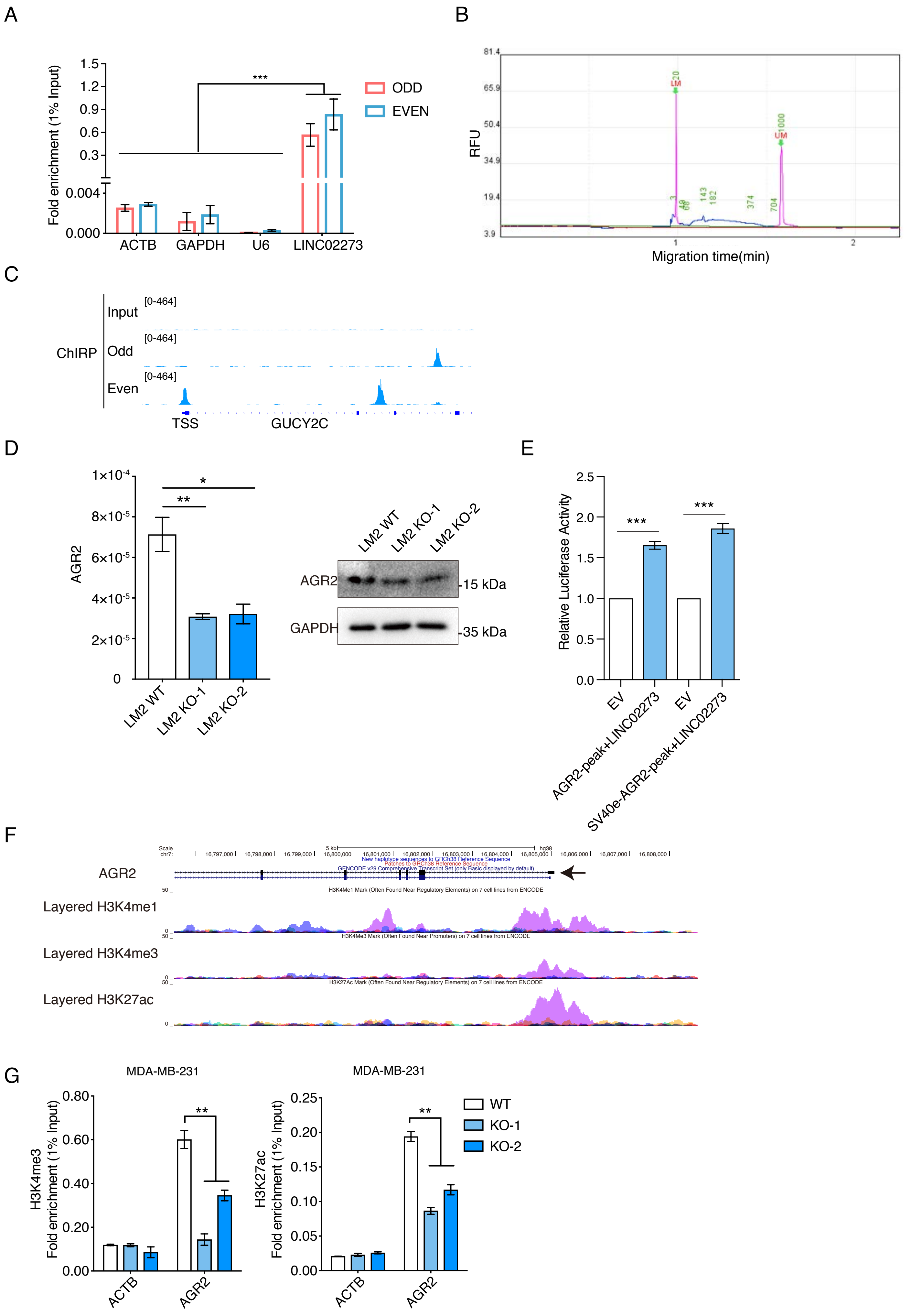
A
B
C
D
E
F
G
Fold enrichment (1% Input)
ODD
EVEN
ACTB
GAPDH
U6
LINC02273
RFU
Migration time(min)
LM
UM
ChIRP
Input
Odd
Even
[0-464]
TSS
GUCY2C
AGR2
LM2 WT
LM2 KO-1
LM2 KO-2
GAPDH
15 kDa
35 kDa
Relative Luciferase Activity
EV
AGR2-peak+LINC02273
SV40e-AGR2-peak+LINC02273
Layered H3K4me1
Layered H3K4me3
Layered H3K27ac
MDA-MB-231
H3K4me3
H3K27ac
WT
KO-1
KO-2

Figure S6

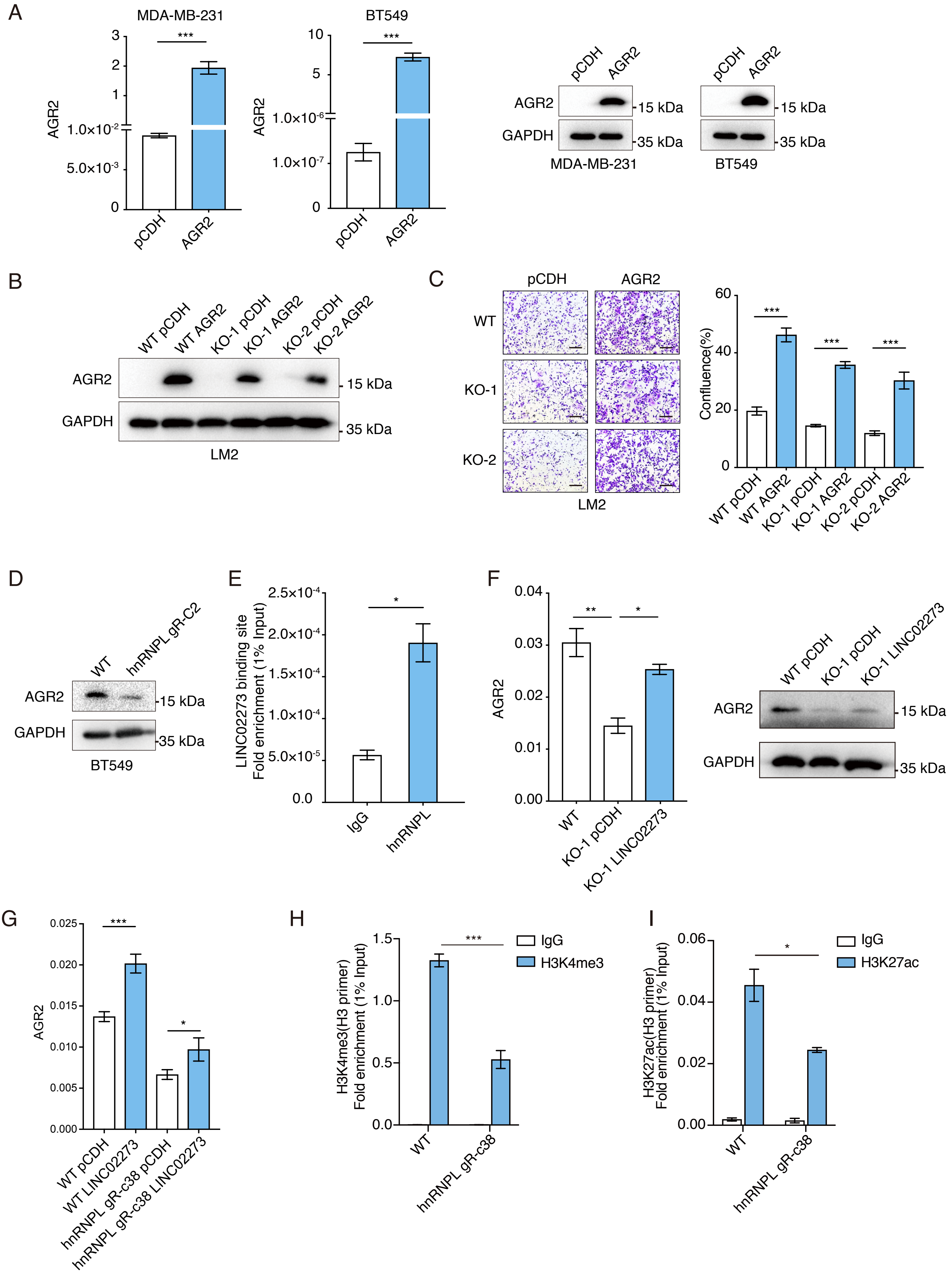
A
MDA-MB-231
AGR2
pCDH
AGR2
BT549
AGR2
GAPDH
15 kDa
35 kDa
B
WT pCDH
WT AGR2
KO-1 pCDH
KO-1 AGR2
KO-2 pCDH
KO-2 AGR2
LM2
C
WT
KO-1
KO-2
Confluence(%)
D
hnRNPL gR-C2
E
LINC02273 binding site
Fold enrichment (1% Input)
IgG
hnRNPL
F
KO-1 LINC02273
G
WT LINC02273
hnRNPL gR-c38 pCDH
hnRNPL gR-c38 LINC02273
H
H3K4me3(H3 primer)
Fold enrichment (1% Input)
H3K4me3
hnRNPL gR-c38
I
H3K27ac(H3 primer)
H3K27ac

Figure S7

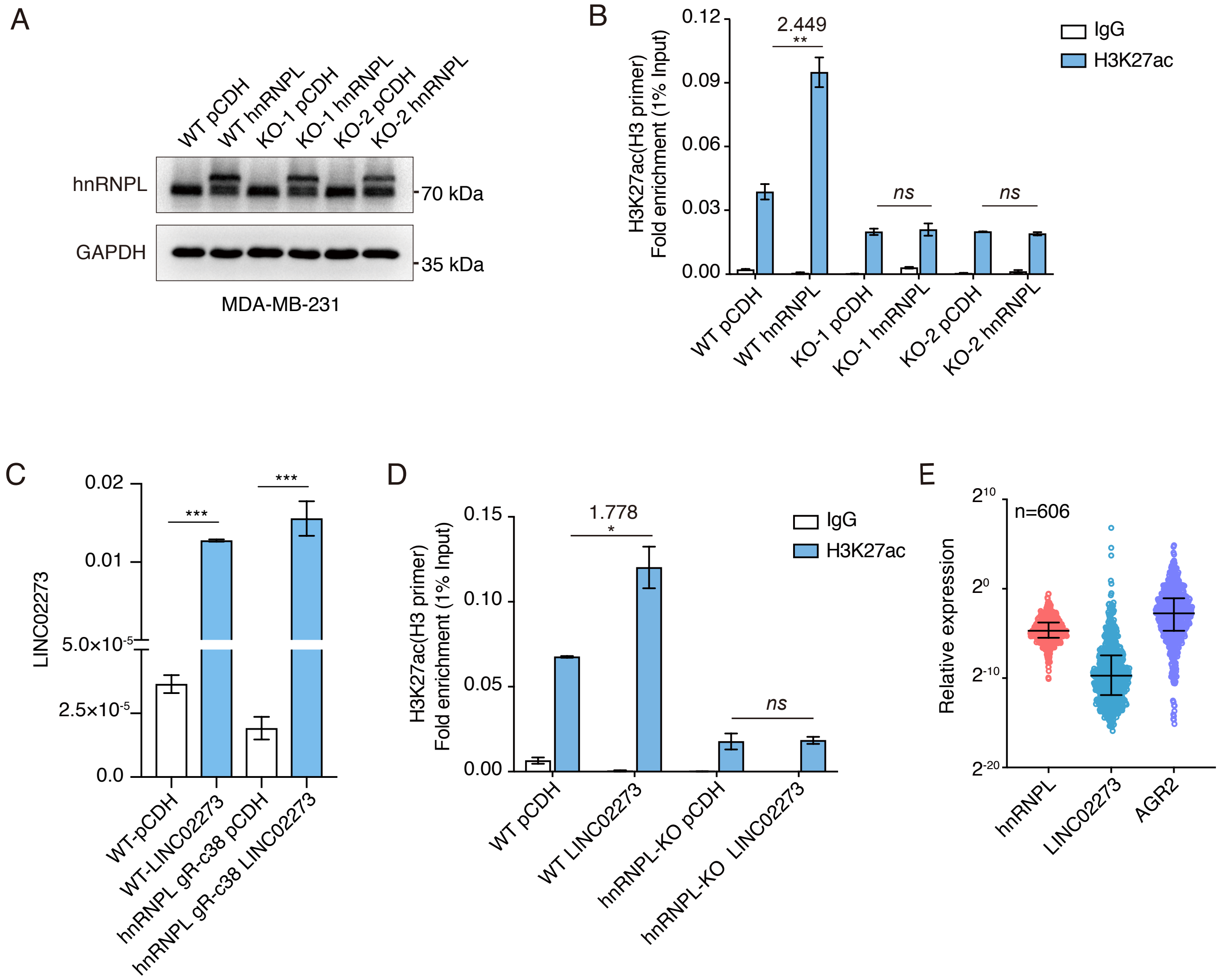

A
WT pCDH
WT hnRNPL
KO-1 pCDH
KO-1 hnRNPL
KO-2 pCDH
KO-2 hnRNPL
hnRNPL
70 kDa
GAPDH
35 kDa
MDA-MB-231
B
H3K27ac(H3 primer)
Fold enrichment (1% Input)
0.12
0.09
0.06
0.03
0.00
2.449
**
ns
ns
IgG
H3K27ac
WT pCDH
WT hnRNPL
KO-1 pCDH
KO-1 hnRNPL
KO-2 pCDH
KO-2 hnRNPL
C
LINC02273
0.02
0.01
5.0×10-5
2.5×10-5
0.0
***
***
WT-pCDH
WT-LINC02273
hnRNPL gR-c38 pCDH
hnRNPL gR-c38 LINC02273
D
H3K27ac(H3 primer)
Fold enrichment (1% Input)
0.15
0.10
0.05
0.00
1.778
*
ns
IgG
H3K27ac
WT pCDH
WT LINC02273
hnRNPL-KO pCDH
hnRNPL-KO LINC02273
E
Relative expression
2^10
2^0
2^-10
2^-20
n=606
hnRNPL
LINC02273
AGR2

Figure S8

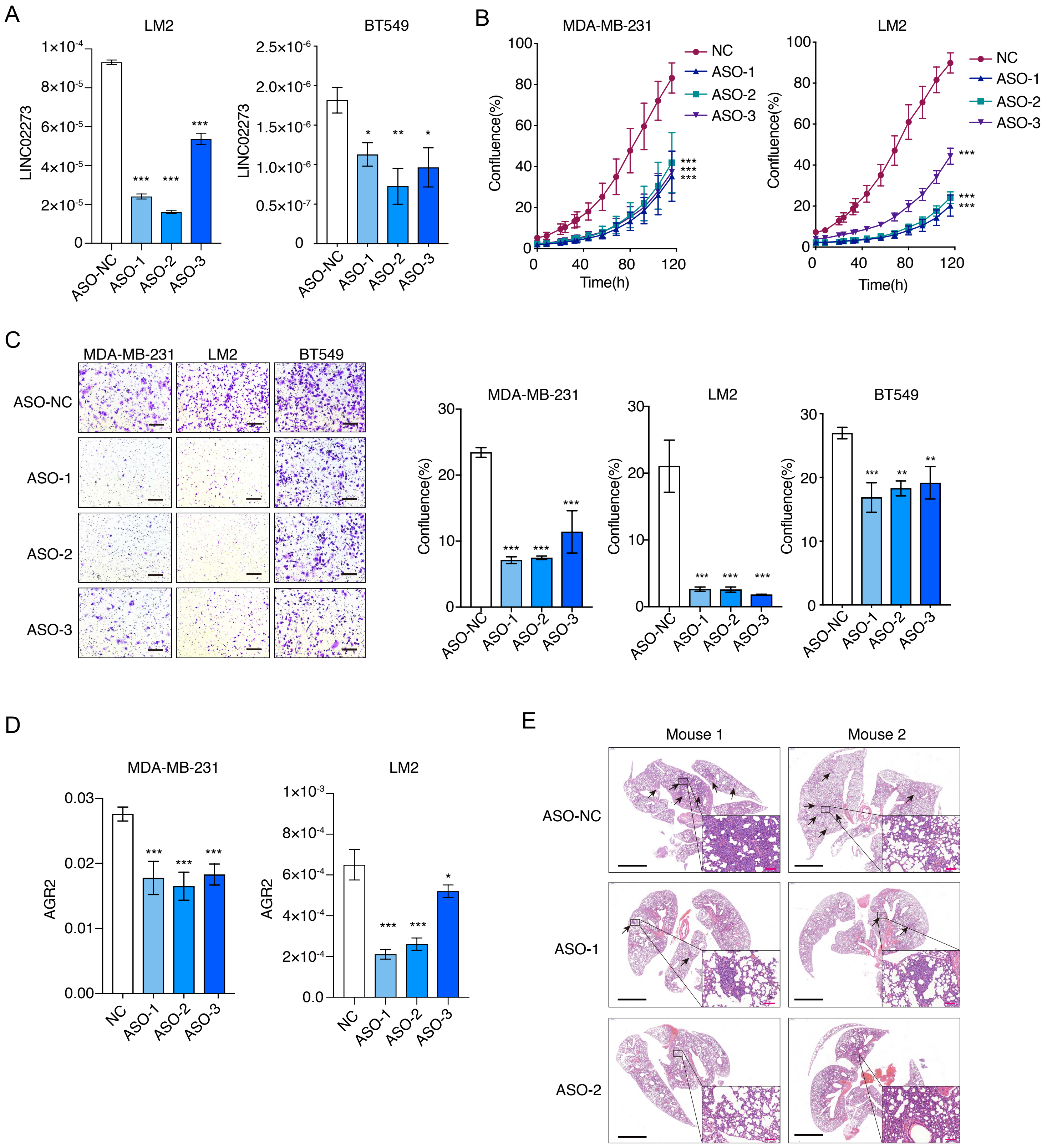
A
LM2
BT549
LINC02273
ASO-NC
ASO-1
ASO-2
ASO-3
B
MDA-MB-231
LM2
Confluence(%)
Time(h)
NC
ASO-1
ASO-2
ASO-3
C
MDA-MB-231
LM2
BT549
ASO-NC
ASO-1
ASO-2
ASO-3
Confluence(%)
D
MDA-MB-231
LM2
AGR2
NC
ASO-1
ASO-2
ASO-3
E
Mouse 1
Mouse 2
ASO-NC
ASO-1
ASO-2

Additional file 1: Table S1. Patient characteristics of RNA microarray

Patient	Tumor Size	Grade	ER	PR	HER2	Ki-67	SLN (+)	non-SLN (+)
Case A	1.7 cm	II	100%+	90%+	IHC-	20%	1	0
Case B	2 cm	II	90%+	90%+	IHC-	40%	2	0
Case C	2.2 cm	III	70%+	70%+	IHC-	30%	1	0
Case D	1.8 cm	II	90%+	5%+	FISH-	20%	1	0
Case E	2 cm	II	90%+	90%+	IHC-	40%	2	0

Abbreviations: ER, estrogen receptor; PR, progesterone receptor; HER2, human epidermal growth factor receptor 2; SLN, sentinel lymph node; IHC, immunohistochemistry; FISH: Fluorescence in situ hybridization.

Additional file 1: Table S2. CPAT prediction of LINC02273

Refence genome	RNA Size	ORF Size	Ficket Score	Hexamer Score	Coding Probability	Coding Label
hg19	1653	165	0.5037	-0.15946054	0.00277711	no

Additional file 1: Table S3. CPC prediction of LINC02273

Refence genome	Coding/non coding	Coding potential score
hg19	noncoding (weak)	-0.941269

Additional file 1: Table S4. Baseline clinicopathological characteristics of patients according to LINC02273 expression

Characteristics		Low	High	*P* value
Age at diagnosis	<40	11	8	0.814
	≥40	158	142	
BMI at diagnosis	<18.5	7	1	0.147
	18.5-24	104	98	
	>24	55	51	
Subtype	ER+/PR+	120	118	0.187
	HER2 positive	29	16	
	TNBC	14	16	
ER	Negative	55	36	0.135
	Positive	114	112	
PR	Negative	36	27	0.322
	Positive	118	122	
HER2	Negative	104	101	0.079
	Positive	57	35	
Ki-67	Low	60	57	0.084
	High	53	30	
Tumor grade	I & II	102	88	0.638
	III	58	56	
pT	pT1	69	51	0.369
	pT2	92	94	
	pT3	7	5	
pN	pN0	73	60	0.544
	pN1	52	41	
	pN2	22	20	
	pN3	22	28	
LVI	Negative	89	82	0.496
	Positive	79	62	

Abbreviations: BR: Breast Cancer; HR: hazard ratio; CI: confidence interval; BMI, body mass index; ER, estrogen receptor; PR, progesterone receptor; HER2, human epidermal growth factor receptor 2; LVI: lymphovascular invasion. Fisher exact test was used. Statistically significant (*P<0.05 and **P<0.01).

Additional file 1: Table S5. Univariant COX regression analyses of RFS in BC patients

Characteristics		No of Patients	No of Events	*P* value	HR (95%CI)
Age at diagnosis	<40	13	6		
	≥40	216	84	0.888	0.942 (0.412-2.157)
BMI at diagnosis	<18.5	5	3		
	18.5-24	146	56	0.519	0.682 (0.214-2.179)
	>24	76	30	0.594	0.724 (0.221-2.373)
Subtype	ER+/PR+	171	67		
	HER2	34	11	0.748	0.901 (0.476-1.705)
	TNBC	20	10	0.437	1.302 (0.67-2.531)
ER	Negative	67	24		
	Positive	162	64	0.842	1.049 (0.656-1.677)
HER2	Negative	156	49		
	Positive	64	28	0.217	1.339 (0.842-2.131)
Ki-67	Low	86	31		
	High	51	27	0.135	1.472 (0.887-2.443)
Tumor grade	I & II	143	47		
	III	78	36	0.139	1.388 (0.899-2.144)
pT	pT1	91	29		
	pT2	129	57	0.180	1.357 (0.868-2.123)
	pT3	8	4	0.406	1.558 (0.548-4.432)
pN	pN0	112	21		
	pN1	65	28	0.013*	2.053 (1.166-3.615)
	pN2	28	14	0.015*	2.321 (1.18-4.566)
	pN3	24	26	0.000**	4.355 (2.447-7.75)
LVI	Negative	131	40		
	Positive	96	45	0.076	1.47 (0.96-2.252)
LINC02273	Low	132	37		
	High	97	53	0.016*	1.677 (1.102-2.553)

Abbreviations: BR: Breast Cancer; HR: hazard ratio; CI: confidence interval;BMI, body mass index; ER, estrogen receptor; PR, progesterone receptor; HER2, human epidermal growth factor receptor 2; LVI: lymphovascular invasion. Statistically significant (**P*<0.05 and ***P*<0.01).

Additional file 1: Table S6. Multivariant COX regression analyses of RFS in BC patients

Characteristics		No of Patients	No of Events	*P* value	HR
LINC02273	Low	132	37		
	High	97	53	0.045*	1.543 (1.010-2.358)
pN	pN0	112	21		
	pN1	65	28	0.013*	2.053 (1.166-3.616)
	pN2	28	14	0.018*	2.256 (1.146-4.439)
	pN3	24	26	0.000**	4.171 (2.340-7.434)

Abbreviations: BR: Breast Cancer; HR: hazard ratio; CI: confidence interval; Statistically significant (*P<0.05 and **P<0.01).

Additional file 1: Table S7. LINC02273 knockout neckties.

KO cell line	CRISPR neckties (hg38)	KO length (bp)
LM2 KO-1	chr4:152,100,707-152,101,737	1030
LM2 KO-2	chr4:152,100,707-152,101,737	1030
BT549 KO	chr4:152,100,517-152,101,753	1236
MDA-MB-231 KO-1	chr4:152,100,704-152,101,603	899
MDA-MB-231 KO-2	chr4:152,100,707-152,101,737	1030

Additional file 1: Table S8. MS result of LINC02273 RNA pull-down

	Reference	Count	Unique Peptide Count	Cover (%)	MW	PI
1	HNRNPL	30	12	17.15	64.1	8.46
2	Human IgG H chain	20	10	32.62	50.9	7.89
3	ALB	10	8	11.75	69.2	5.99
4	HRNR	8	8	3.93	282.4	10.05
5	EEF1A1	28	5	22.51	50.1	9.1
6	PKM2	5	5	16.62	37.3	8.47
7	HEL113	6	4	15.45	53.7	5.06
8	HSP70	5	4	18.07	51.9	5.35
9	Actin	5	4	20.55	28.2	5.2
10	HSP60	4	4	11.88	60.0	5.59

Abbreviations: MS: mass spectrometry; MW: molecular weight; PI: isoelectric point;

Additional file 1: Table S9. Baseline clinicopathological characteristics of patients according to AGR2 expression

Characteristics		Low	High	*P* value
Age at diagnosis	<40	15	4	0.606
	≥40	216	84	
BMI at diagnosis	<18.5	7	1	0.746
	18.5-24	145	57	
	>24	77	29	
Subtype	ER+/PR+	158	80	0.000**
	HER2 positive	39	6	
	TNBC	28	2	
ER	Negative	82	10	0.000**
	Positive	148	78	
PR	Negative	68	11	0.001**
	Positive	151	74	
HER2	Negative	147	59	0.576
	Positive	69	23	
Ki-67	Low	89	28	0.736
	High	65	18	
Tumor grade	I & II	137	53	0.593
	III	86	28	
pT	pT1	85	35	0.431
	pT2	138	48	
	pT3	7	5	
pN	pN0	94	39	0.913
	pN1	68	25	
	pN2	32	10	
	pN3	37	13	
LVI	Negative	125	46	0.704
	Positive	100	41	

Abbreviations: BR: Breast Cancer; HR: hazard ratio; CI: confidence interval; BMI, body mass index; ER, estrogen receptor; PR, progesterone receptor; HER2, human epidermal growth factor receptor 2; LVI: lymphovascular invasion. Fisher exact test was used. Statistically significant (*P<0.05 and **P<0.01).

Additional file 1: Table S10. Multivariant COX regression analyses of RFS in BC patients

		No of Patients	No of Events	*P* value	HR
LINC02273/AGR2	low/low	105	27		
	high/low	69	30	0.303	1.317 (0.779-2.227)
	low/high	27	10	0.402	1.365 (0.66-2.826)
	high/high	28	23	0.001**	2.528 (1.436-4.45)
pN	pN0	112	21		
	pN1	65	28	0.011*	2.053 (1.166-3.616)
	pN2	28	14	0.015*	2.256 (1.146-4.439)
	pN3	24	26	0.000**	4.171 (2.340-7.434)

Abbreviations: BR: Breast Cancer; HR: hazard ratio; CI: confidence interval; Statistically significant (*P<0.05 and **P<0.01).

Additional file 1: Table S11. Probes used in this study

FISH probes	Sequences
LINC02273-1	CACCTGAGACACTTTCCA
LINC02273-2	GTTGTGTGCCACTCAGAT
LINC02273-3	CCAGCACACACCACATTT
LINC02273-4	TACTCTCGAGCTCCTCAG
LINC02273-5	CCTTGCCACCTCTTTAAA
LINC02273-6	CTCACTTCCTCACACCAA
LINC02273-7	GGTGCCCTTGACTTTGAA
LINC02273-8	TGTGCCAAGCTTGTTCTC
LINC02273-9	ATCTGGTTTCCTGGTAGC
LINC02273-10	CGGAATGCAGTTTCCCAT
LINC02273-11	TTCCTATACTTCCCTGGA
LINC02273-12	GGTCTTCCCACTTTTATT
LINC02273-13	GCAGCTGGATGACTGCAA
LINC02273-14	GTGAGCCCTCGAGAGAAA
LINC02273-15	TTCACATCCTCCCAGGAC
LINC02273-16	TGGGAGTCAGGGTACAGG
LINC02273-17	AGACTCTTGGCCTCAGTG
LINC02273-18	GAGTCCTAGAGGACAGCT
LINC02273-19	GTGGCTGAAGTGAGTGAA
LINC02273-20	GAAACACAGTCAGCGCTT
LINC02273-21	CAGGAACTGCCCTTTGGT
LINC02273-22	AGAGGGGGAGTAGAAGCC
LINC02273-23	AGTAACCCTGGCACCTTG
LINC02273-24	AGTGATGCCATGGAAGGC
LINC02273-25	AAACCATCCAGGCTCACT
LINC02273-26	GGGTTTTTTGTTGTCGGC
LINC02273-27	CCTTGGGGAAGGGGAATG
LINC02273-28	CCTTGCCCTGGGTAAGTG
LINC02273-29	ACTCAAATCTCAGGCTGG
LINC02273-30	CCTGTAGGGGACTCAGTA
LINC02273-31	TTCAGCTCATAGCTGTGC
LINC02273-32	CTGAGGTCTCAGGGGTTA
LINC02273-33	GTGTCCAAATACTGTCCC

LINC02273-34	GGATCCCCTCAGTAAACT
LINC02273-35	CTTCCCACTGCACACTAA
LINC02273-36	TTGTGGATGGCCAAGCTT
LINC02273-37	GCCCAAGATCAAGGTGTC
LINC02273-38	TGGCATGCTGTCCACAGA
LINC02273-39	CAGGGATAACTCGTGCGA
LINC02273-40	CAGCCTGCTAATTTGAGC
LINC02273-41	CTGAAGCCTGTCCAGGAA
LINC02273-42	TGTCTGTATCACCAGGGA

ChIRP probes	Sequences
PROBE-LINC02273-1	ACACTTTCCAAGCATTGCAA
PROBE-LINC02273-2	AGCACACACCACATTTCATA
PROBE-LINC02273-3	ACTTCCTCACACCAATCAAG
PROBE-LINC02273-4	CCAGACACACTGGACTTATT
PROBE-LINC02273-5	ATAATAGGTCTTCCCACTTT
PROBE-LINC02273-6	TGATGGGAGTCAGGGTACAG
PROBE-LINC02273-7	GTGGCTGAAGTGAGTGAACT
PROBE-LINC02273-8	AGACAGTGATGCCATGGAAG
PROBE-LINC02273-9	ACTCAAATCTCAGGCTGGAC
PROBE-LINC02273-10	AATGAACCTGGAGAGAGGCA
PROBE-LINC02273-11	GTTGTATTGGATGAGGATCC
PROBE-LINC02273-12	ATTTGTTCTGAGTCTCTCTC
PROBE-LINC02273-13	CCGTGACAAAGGACCACAGG
PROBE-LINC02273-14	CATGCTGTCCACAGACAGTG
PROBE-LINC02273-15	GTATCACCAGGGAGAATGTT
PROBE-LACZ-1	AAAATAATTCGCGTCTGGCC
PROBE-LACZ-2	GCGTTAAAGTTGTTCTGCTT
PROBE-LACZ-3	TCGGCAAAGACCAGACCGTT
PROBE-LACZ-4	CGCATCAGCAAGTGTATCTG

Additional file 1: Table S12. Primers used in this study

RT-qPCR primers	Sequences
qU6-S	CTCGCTTCGGCAGCACATAT
qU6-AS	TATGGAACGCTTCACGAATTTG
qGAPDH-S	AACGGGAAGCTTGTCATCAA
qGAPDH-AS	TGGACTCCACGACGTACTCA
qACTB-S	GCCAACCGCGAGAAGATGA
qACTB-AS	CATCACGATGCCAGTGGTA
qhnRNPL-S	TACGCAGCCGACAACCAAATA
qhnRNPL-AS	CTCCGGGAGTCATCCGAGT
qMYC-S	AGCTGCTTAGACGCTGGATTT
qMYC-AS	CGAGGTCATAGTTCCTGTTGGT
qLINC02273-1653-S	CTCCCAGCAGCTAAGGTGAC
qLINC02273-1653-AS	CACTTCCTCACACCAATCAAGTC
qLINC02273-1252-S	CTCCCAGCAGCTAAGGTAGAAG
qLINC02273-1252-AS	TACAGGGTTCACATCCTCCCA
qFOXN4-S	AGGGCTCCTGTAGACTTCATC
qFOXN4-AS	CCAAGCTGAATCCCTCATCCT
qGABRG1-S	GTAAACAGCATTGGACCAGTTGA
qGABRG1-AS	CGAAGCAGACGATTAGGAGTTG
qZNF831-S	CAGACGCACCTCAACAACTC
qZNF831-AS	GCTTCGGTCCTCACATCCAG
qCHRNA7-S	GCTGGTCAAGAACTACAATCCC
qCHRNA7-AS	CTCATCCACGTCCATGATCTG
qEBF1-S	TGGGGTTCGTGGAGAAGGAA
qEBF1-AS	CACGTAGAAATCCTGCTCCG
qERP27-S	CCATACTCCATAGCATGGTGC
qERP27-AS	TGATGTTGTAGTGTGTCAGAACC
qGUCY2C-S	CCTTGACACAGAATTGAGCTACC
qGUCY2C-AS	CATCAGCCTGGTTAAGGTTTCTT
qAGR2-S	AGAGCAGTTTGTCCTCCTCAA
qAGR2-AS	CAGGTTCGTAAGCATAGAGACG

ChIP-qPCR & ChIRP-qPCR	Sequences
qdnFOXN4-S	TGGGCTGTTGGTGAGTGAAC
qdnFOXN4-AS	CCAGCAACCGTGAGTGGTT
qdnGABRG1-S	AGCTCAACTCTCTTCCTGTTGTT
qdnGABRG1-AS	TGGGATTTCCCCTCAAGGCATTT
qdnZNF831-S	GTTAAATGACTGCTGAGCCTCCA
qdnZNF831-AS	GCTCACAGGTGTTCTCACTTGG
qdCHRNA7-S	GGTAATTGCAGGTTGCCTCTGA
qdCHRNA7-AS	AACAGCTGGGGTTTTTGATCTT
qdnEBF1-S	TGAAATGTGGTTGGGTGCTTAT
qdnEBF1-AS	CATACTGCAATCAAATCCGGCA
qdnERP27-S	CCTGAAGGCATTAGAGAGCCAG
qdnERP27-AS	AAAAGCAGTCCTCAATGCAGGG
qdnGUCY2C-S	CCAAAACCAGGGATGAGATGGT
qdnGUCY2C-AS	TGAAGGGAGAGGGCAAGTTTCA
qdnAGR2-S	AAAAACCCCTCCTTTGCTTGTCT
qdnAGR2-AS	TTGTCATAGTGGCTGACGGATG
qdnH3-S	CTGAGACGAAAATCCCTGGACC
qdnH3-AS	CTTTCTTTGGCTAGAGCTGGCA

Race primers	Sequences
3'RACE	ACTCTGGGTGGTCCTGGGAGGATGTGAA
5'RACE	TTAGGGGACTCAGTAGGAACTCAAATCTCAGG

KO genotyping primers	Sequences
LINC02273KO-S	GAAAGCAGAGAAAAATTCATCAGACACGG
LINC02273KO-AS	AGGGTCCTTTCAGTATTTTTCCACAGG

Additional file 1: Table S13. shRNA, siRNA, CRISPR, and ASOs

shRNA target sequence	
shLINC02273-2	GTGGGAAGACCTATTATAAGT
shLINC02273-3	CTCTCCAAGGTGCCAGGGTTA
shSCR	CCTAAGGTTAAGTCGCCCTCG

CRISPR target sequence	
LINC02273KO-S1-F	GGCAACAAGTGTGAAGGGTGA
LINC02273KO-S2-F	GCAGGAAGTGGGTGTACATGT
LINC02273KO-AS1-F	GGGGGATACTGGCTGAAAACA
LINC02273KO-AS2-F	GGTGTTAGGCCTGAGAGATGA
hnRNPL-1	TCTGAAGATCGAATACGCAA
hnRNPL-2	CTGGGGACTCGGATGACTCC

siRNAs target sequence	
sihnRNPL1	CATCATGCCTGGTCAGTCA
sihnRNPL2	AGGTTTGTAGAGGCTTACT
siNC	TTCTCCGAACGTGTCACGT

ASOs sequence	
ASO-1	CTCTCCAGGTTCATTCCTAA
ASO-2	ACAAATCAATCTGCGGACAC
ASO-3	CACACACCCAAGAAAACAAA
NC	lnc6N0000001, Ribobio
